# Generative models, linguistic communication and active inference

**DOI:** 10.1016/j.neubiorev.2020.07.005

**Published:** 2020-11

**Authors:** Karl J. Friston, Thomas Parr, Yan Yufik, Noor Sajid, Catherine J. Price, Emma Holmes

**Affiliations:** a The Wellcome Centre for Human Neuroimaging, UCL Queen Square Institute of Neurology, 12 Queen Square, London, WC1N 3AR, UK; b Virtual Structures Research, Inc., 12204 Saint James Rd, Potomac, MD 20854, USA

**Keywords:** Message passing, Inference, Connectivity, Free energy, Neuronal, Hierarchical, Bayesian, Language

## Abstract

This paper presents a biologically plausible generative model and inference scheme that is capable of simulating communication between synthetic subjects who talk to each other. Building on active inference formulations of dyadic interactions, we simulate linguistic exchange to explore generative models that support dialogues. These models employ high-order interactions among abstract (discrete) states in deep (hierarchical) models. The sequential nature of language processing mandates generative models with a particular factorial structure—necessary to accommodate the rich combinatorics of language. We illustrate linguistic communication by simulating a synthetic subject who can play the ‘Twenty Questions’ game. In this game, synthetic subjects take the role of the questioner or answerer, using the same generative model. This simulation setup is used to illustrate some key architectural points and demonstrate that many behavioural and neurophysiological correlates of linguistic communication emerge under variational (marginal) message passing, given the right kind of generative model. For example, we show that theta-gamma coupling is an emergent property of belief updating, when listening to another.

## Highlights

• New (hierarchical) generative model for linguistic interactions.
• Builds on active inference formulations of dyadic interactions.
• We simulate agents that ask and answer questions together.
• Theta-gamma coupling emerges from belief updating under this framework.

## 1 Introduction

In April 2018, an international group of experts assembled in Frankfurt for an Ernst Strüngmann forum addressing complexity and computation in the cortex (Singer et al., 2019). One group was briefed to discuss human cognition and, reflecting the interests of that group, chose to focus on language processing—a challenging area for computational neuroscience, linguistics and theoretical neurobiology (Hauser et al., 2002). What follows is a formal analysis that speaks to a key conclusion of that group; namely, that the neuronal correlates of language processing and functional brain architectures should emerge naturally, given the right kind of generative model. In brief, this paper uses simulations of linguistic communication to show that many behavioural and neurophysiological correlates of language processing emerge under deep diachronic models.

A generative model refers to a probabilistic mapping from causes (e.g., semantics) to consequences (e.g., auditory signal). Perception, recognition or inference then becomes the (Bayesian) inversion of the generative model to infer causes from consequences. The notion of a generative model rests on a commitment to the brain as a constructive organ, generating explanations for its sensations. We will use an active inference (a.k.a., predictive processing) formulation of this basic idea that inherits from a long tradition of psychological ideas about how the brain works; from Kant through Helmholtz (Helmholtz, 1878 (1971)), from analysis by synthesis (Yuille and Kersten, 2006) to perception as hypothesis testing (Gregory, 1980), from the Helmholtz machine (Dayan et al., 1995) to the free energy principle (Friston, 2010). Specifically, we will use a corollary of the free energy principle; namely, active inference (Friston et al., 2017a). The basic idea behind active inference is that any neuronal processing can be formulated, in a normative sense, as a minimisation of the same quantity used in approximate Bayesian inference; i.e., a variational free energy or evidence bound (Mattys et al., 2005; Winn and Bishop, 2005).

Minimizing variational free energy is equivalent to maximizing the sensory evidence for an internal model of how unobserved (i.e., hidden) states of the world generate observed (i.e., sensory) consequences. Technically, this can be formulated in terms of maximising the marginal likelihood for models of the lived world—that is neatly summarized as self-evidencing (Clark, 2016; Hohwy, 2016); in other words, gathering sensory evidence for our generative models. Having specified the generative model one can then use standard, ‘off-the-shelf’ belief updating schemes (Friston et al., 2017c) to create synthetic agents, who perceive and act in a self-evidencing fashion. These simulations can also be used to predict empirical behavioural and physiological responses. Here, we use simulations to test hypotheses about communication; such as generation and understanding of linguistic phrases, in relation to conceptual knowledge (Barsalou, 2003; Yufik, 1998, 2019), the use of a shared narrative (Mar et al., 2011; Mathewson et al., 2019), and the linearization of language (Bornkessel et al., 2005).

This paper extends a long line of existing work in the domain of natural language processing (and response generation). Previously, the focus has been on treating natural language processing as a *learning* problem (Elman, 1990), where the use of deep learning has spearheaded algorithmic developments (Young et al., 2018): e.g., word embeddings derived from learning predictive relationships (Collobert et al., 2011; Mikolov, 2010; Mikolov et al., 2013; Pennington et al., 2014) and fully contextualized word representations (Devlin et al., 2018; Radford et al., 2019; Vaswani et al., 2017). These approaches to natural language processing limit themselves to learning associations—between an input and output—via the training of particular neural networks. In contrast, response generation—including conversational dialogue agents—have been framed as either *deep reinforcement learning* (Li et al., 2016; Zhao et al., 2017) or *inference* problems (Liu et al., 2018). These approaches, whilst closely aligned with our work, are optimising objective functions that do not account for the future, including their ability to have forward-looking conversations, due to word-repetitions or closed-form replies (Li et al., 2016). In contrast, by framing language as an active (Bayesian) inference problem, with an underlying generative model, our approach infers causal relationships between inputs and outputs—and provides a structural understanding of the sequences of words being presented and their context sensitivity. This results in uncertainty resolving actions that lead to forward-looking conversations: as demonstrated in the simulations that follow, an agent does not need to revisit issues that have already been resolved.

The resulting approach also differs from previous cognitive theories of language processing. Although the idea of ‘surprisal’ has become increasingly prevalent in the literature (Hale, 2001; Levy, 2008), this usually refers to the magnitude of ‘surprise’ conveyed by individual words, such that expected semantics are simply an amalgamation of the semantics conveyed by all preceding words. In contrast, in the current formulation, belief updating occurs at a higher level and relies on beliefs about an acoustic scene, about which the agent has prior beliefs. Note that the mathematical formulation used here—which is described in detail in the sections that follow—differs from previous approaches in this literature. There are two key points to note here. First, the current formulation considers the uncertainty of the agent’s beliefs about the scene at hand. Second, we introduce an active component—which generates predictions about the information that an agent will seek to resolve their uncertainty. In other words: What questions should I ask next, to resolve my uncertainty about the subject of our conversation?

This paper comprises four sections. The first (Generative models of language) describes a top-down approach to understanding functional brain architectures in terms of generative models, with a special focus on models that are apt for linguistic communication. This section considers the requisite computational architecture and the second section (Active inference) describes the accompanying message passing. The third section (“Twenty Questions” simulations) uses the generative model to illustrate behavioural and neurophysiological correlates of speaking and listening (Edwards and Chang, 2013; Kayser, 2019; Lizarazu et al., 2019; Pefkou et al., 2017). This section concludes with a demonstration of how the model predicts responses that would be interpreted as theta-gamma coupling (Giraud and Poeppel, 2012; Lizarazu et al., 2019; Pefkou et al., 2017). It also reproduces some simple violation paradigms, in terms of synthetic event related potentials and difference waveforms—of the sort seen in mismatch negativity, P300, and N400 studies (Coulson et al., 1998; Van Petten and Luka, 2012). The final section (Synthetic communication) turns to communication *per se*, using dyadic interactions between two synthetic subjects to illustrate that certain kinds of belief updating can be instantiated linguistically. We conclude with a discussion of what has not been addressed; for example, a sense of agency and the acquisition of language through learning deep models.

## 2 Generative models of language

Before modelling linguistic communication, we first begin with a simplified generative model of how spoken phrases are generated by an individual synthetic agent. This generative model is not intended to be a comprehensive model of language, but rather specify key components of a computational architecture that will allow us to simulate linguistic communication. The advantage of focusing on generative models—as opposed to recognition models—is that the same generative model can be used to generate an auditory signal given a narrative (i.e., for language production) and to infer the narrative given auditory input (i.e., for language understanding). Here, we focus on simulating a simple agent, who can ask questions and answer them. In this formulation, the agent does not know whether its beliefs are its own or are generated by some external narrator. We will return to this issue in the discussion.

So, what are the special requirements of a generative model for language? Here, we take a common-sense approach and list the necessary properties such a model must possess. Starting with the generative model somewhat simplifies things, in the sense that one only has to specify what would be sufficient to generate meaningful language. One can then simulate basic language understanding by applying established inversion schemes. First, we will assume that language is for communication, which immediately implies a shared forward-looking narrative (Allwood et al., 1992; Brown and Brune, 2012; Friston and Frith, 2015a; Mar et al., 2011; Schegloff and Sacks, 1973; Specht, 2014). In turn, this implies shared (and evolving) beliefs about the subject of communication (Mathewson et al., 2019). This simple observation has some fundamental implications. The first may be slightly counterintuitive and borrows from earlier work on neuronal hermeneutics (Friston and Frith, 2015a). This work—using generalised synchrony to simulate communication between songbirds—suggests that it is sufficient to share the same generative model to infer the meaning of sensory exchanges between interlocutors. The issue of who is talking and attribution of agency then becomes a somewhat secondary issue, which is only necessary for turn-taking (Ghazanfar and Takahashi, 2014; Wilson and Wilson, 2005). In short, a narrative cannot be uniquely attributed to you or me—it is *our* narrative.

The notion of a shared narrative is central to our formulation of the generative model. Usually, in realising or simulating active inference (in real artefacts or *in silico*), outcomes are generated by external states of the world that agents navigate. These sensory outcomes are then used to update beliefs about external states, which are used to plan actions. Policies—which are sequences of actions—change external states and generate new outcomes. And so, the perception-action cycle continues. However, in the context of dyadic exchange, outcomes are generated by another person or agent, *without any necessary reference to external states*. In this setting, when an artificial agent speaks, it generates outcomes that are most consistent with its beliefs which, in turn, update the beliefs of its correspondent. The upshot of this exchange is a synchronisation or alignment of belief states that—in pure communication—circumvent any reference to external states of the world.

**Fig. 1:**
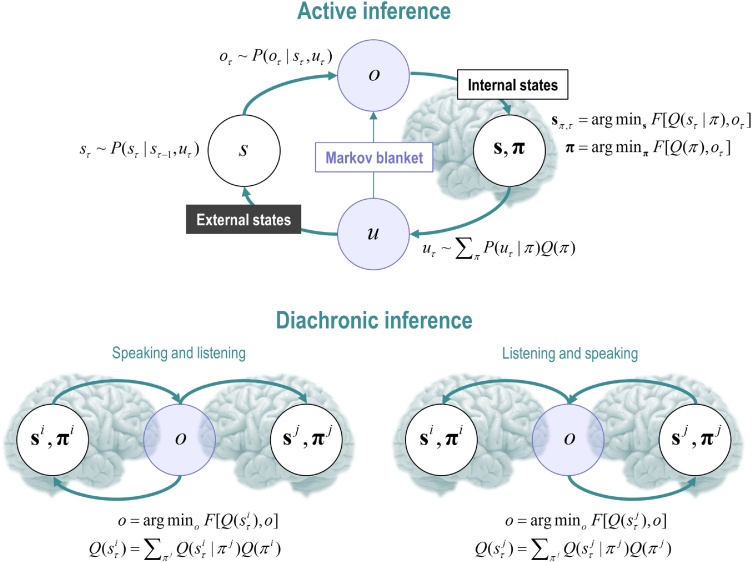
**Active inference and Markov blankets**. This figure illustrates the conditional dependencies among various states that constitute (active) inference about external states of affairs in the world. Active inference rests upon a four-way partition of states into external states (*s*) and internal states (**s**, **π**) that are separated by Markov blanket states (*o*, *u*). Technically, the Markov blanket of internal states comprises their parents, their children and the parents of the children. In this figure, blanket states correspond to the pale blue circles. Blanket states comprise observations or outcomes (*o*) and action (*u*). The upper panel illustrates the standard way in which conditional dependencies are mediated: internal states are treated as encoding representations of external states. These representations prescribe action on external states, which generates outcomes. In this construction, internal states play the role of sufficient statistics or parameters of a posterior belief (*Q*) about external states and plans or policies that are realised by action. These beliefs are optimised by minimising a free energy functional of posterior beliefs, given outcomes. Posterior beliefs about the policies provide a probability distribution from which the next action is sampled. This action changes external states, which generate outcomes – and so the (perception-action) cycle continues. The lower panel shows the simplified scheme used in this paper, labelled ‘Diachronic inference’. In this setting, actions (*u*) and outcomes (*o*) are assumed to be isomorphic. In other words, I act by generating an outcome that minimises free energy. This is equivalent to generating or selecting outcomes that are the most likely under my beliefs about the causes of that outcome. Because these outcomes are shared between two (or more) agents, they constitute the Markov blanket that separates the internal states of every agent in the exchange. This means the internal states of one agent now constitute the external states of another (and *vice versa*). Crucially, this rests upon a diachronic *switching*, in which only one agent generates outcomes at any one time. Heuristically, this means that I can either listen or speak but not both at once. With this particular constraint on conditional dependencies, the shared outcome is (e.g., spoken words) constitute the blanket states that are shared by all agents. The superscripts in the lower panel denote two agents (*i* and *j*). The equations express the sampling of various states, or their minimisation with respect to variational free energy. An interesting aspect of the diachronic setup is that everything minimises a free energy; effectively resolving uncertainty; such that the beliefs of one agent are installed in another, via an exchange of outcomes.

This alignment follows naturally from generating outcomes that are consistent with beliefs (technically, outcomes that have the greatest marginal likelihood or model evidence). Actions and outcomes are assumed to be isomorphic. Subsequent belief updating based on those outcomes makes the beliefs of both subjects consistent with the outcomes they share. In short, outcomes and beliefs are selected in concert to maximise model evidence and, implicitly, the predictability of sensory samples. The inevitable endpoint of this reciprocal exchange is convergence to the same belief states (Isomura et al., 2019), which ensures the outcomes generated by one agent are easily predictable, in virtue of the fact that these are the same outcomes the agent would have produced itself. This kind of generalised synchronisation has been explored in numerical analyses of communication by birdsong and intracellular communication (Friston et al., 2015; Friston and Frith, 2015b; Isomura et al., 2019; Kuchling et al., 2019). In this paper, we focus on pure communication; in the sense that all outcomes are generated by one or another agent. This means there are no other states of the world to consider. See Fig. 1 for a graphical depiction of the special conditional dependencies implied by pure communication.

In what follows, we try to show how the belief states of two or more agents become aligned through pure communication, where this alignment is an emergent property of selecting beliefs that are consistent with what is heard while, at the same time, generating outputs that are consistent with those beliefs. If two or more agents comply with these imperatives, their beliefs align, thereby evincing a minimal form of communication. It is interesting to consider how external states might get into the game; for example, providing visual cues that affect the beliefs of one agent: i.e., how does one person convey her beliefs about what she is seeing to another, or how do they reach consensus when they can see different parts of the same scene? However, in this work, we will just consider pure communication without external states and focus on how beliefs about a scene are installed by a shared narrative.

So, what is a narrative? On the active inference view, everything we do can be regarded as pursuing a narrative that resolves uncertainty (Friston et al., 2017a; Mirza et al., 2016). This means that the only sort of narrative that matters is one that has epistemic affordance; namely, the opportunity to reduce uncertainty under a particular belief structure about the world. In this sense, the formal imperatives for language become exactly the same as any active inference; for example, active vision (Ferro et al., 2010; Ognibene and Baldassarre, 2014). Indeed, the same principles underlie experimental design in scientific enquiry, where one solicits data that disambiguate among competing hypotheses (Lindley, 1956). Much of the motivation for the generative model below inherits from the formally identical problem of querying the world with saccadic eye movements during scene construction (Mirza et al., 2016; Ognibene and Baldassarre, 2014; Purpura et al., 2003). In short, we will regard language as acting on the world to resolve uncertainty, where epistemic foraging has been elevated from visual palpation (i.e., visual search in active vision) to an interrogation of the world via semantics and semiotics, acquired by encultured learning (Constant et al., 2019; Creanza and Feldman, 2014; Penn et al., 2008; Rizzolatti and Craighero, 2004). Following this analogy to its conclusion, a minimal but sufficient model of language for communication can then be framed in terms of a series of ‘questions’ and ‘answers’ (i.e., propositions and responses), in the same way that sampling the world with our sensory epithelia constitutes a ‘question about what is out there’ (Gregory, 1980). And the subsequent sensory samples provide some salient, uncertainty reducing, evidence for our beliefs about the world.

With this in mind, we set ourselves the task of formulating a generative model that could play a game of “Twenty Questions”. In other words, a model that could generate a sequence of questions and closed “yes/no” answers, which progressively reduce uncertainty about the subject of conversation (i.e., contextual knowledge). These sorts of sequential communication games have extensively been tackled in the literature: including one round of question-answer ‘whisky pricing’ interaction (Hawkins et al., 2015), playing restricted ‘cards corpus’ with one-off communication (Potts, 2012), sequential ‘info-jigsaw’ game (Khani et al., 2018), ‘hat game’ where agents learn to communicate via observing actions (Foerster et al., 2016) and conversations about visual stimulus (Das et al., 2017). Having specified the generative model for our “Twenty Questions” paradigm, we made no further assumptions—we used off-the-shelf (marginal) message passing to simulate neuronal processing (Dauwels, 2007; Friston et al., 2017c; Parr and Friston, 2018; Winn and Bishop, 2005). Exactly the same belief updating scheme, for partially observed Markov decision processes, has been used in many contexts; ranging from exploration of mazes and economic game theory, through to abstract rule solving and scene construction (Friston et al., 2017a). We anticipated that these simulations would reproduce key behavioural and neuronal responses seen in empirical language studies.

### 2.1 A deep diachronic model of language for communication

In brief, our generative model has to generate a sequence of questions and answers, under the constraint that they are articulated as a discrete sequence of continuous outcomes; here, spoken words. This means that narratives emerge at several (i.e., discrete and continuous) levels, which speaks to the deep or hierarchical aspects of the requisite model. This way of hierarchically framing the conversational dialogue problem, has previously been explored through the inclusion of two separate (fast and slow) levels using artificial neural networks (George et al., 2017; Serban et al., 2016; Sordoni et al., 2015). To illustrate this deep structure and implicit separation of temporal scales, we considered the problem of generating a succession of question and answers that depend upon beliefs about the world. States of the world come in many flavours. We will refer to these states as hidden *factors*, where each factor (e.g., ‘colour’) has a number of discrete *states* (e.g., ‘red’, ‘green’, ‘blue’ …). The use of factors is known as a *mean field approximation* in the variational machine learning literature (Jaakkola and Jordan, 1998; Kschischang et al., 2001; Sallans and Hinton, 2004; Zhang et al., 2018) and is important for simplifying the form of the generative model and ensuing inference. In fact, the notion of approximate Bayesian inference using variational Bayes, is defined operationally in terms of this sort of factorisation.

The problem of specifying a generative model now reduces to specifying the factors that are sufficient to generate a particular question or answer. These include the form of the question, its content, and the beliefs about the world that determine the correct answer. By inducing a factorisation between the form of the sentence and its content, one can finesse the combinatorics of representing all possible questions with all possible content. In other words, we will assume that the brain represents—at some suitably high level—the form of a question and its content separately, where the two only interact when generating an outcome or context for the hierarchical level below.

In this paper, we consider two hierarchical levels; namely, a *conceptual* level generating *syntax* and *semantics*, and a lower level generating *lexical* sequences of words or phrases. One could consider further levels, all the way down to phonemes and articulation; however, this level of modelling has already been considered in the context of *active listening* (Friston et al., 2020) We will therefore restrict the current analyses to the generation and understanding of fully formed words (noting that the Matlab simulations that accompany this paper include a full three-level demonstration that supports spoken answers and questions: please see software note).

So, what does one need to know to generate a sentence? Basically, we need the temporal structure or *syntax* of the question, the *semantic* content—that fills in content words like nouns and verbs—and the answer (e.g., ‘yes’ or ‘no’). However, to generate syntax and semantics, we need the narrative (e.g., is this a question or answer?) and the form of the question (e.g., is this a question about *where* something is—i.e., location—or *what* something is—i.e., shape?). We also need to know the states of the world being described (e.g., contextual or scenic knowledge) and which particular attributes are being discussed. These conceptual constructs constitute the highest level of the generative model; namely, everything one would need to know to specify the syntax and semantics of a lower-level.

In deep models of this sort, deeper hierarchical levels are constituted by factors that change over progressively longer timescales (Friston et al., 2017d; George and Hawkins, 2009; Kiebel et al., 2009). This means higher level factors are attributes of a sentence or phrase, while lower level factors may change from word to word (Chien and Honey, 2020; Davis and Johnsrude, 2003; Demirtaş et al., 2019; Specht, 2014; Stephens et al., 2013). Here, high-level factors include the *narrative* structure; namely, is this sentence a prompt, question or answer? If this sentence is a *question*, then what is the question about; e.g., the location or colour of an object? If the narrative requires an answer, then there have to be *scenic* factors encoding states of the world that render the answer right or wrong; e.g., the object is ‘red’. Finally, and possibly most importantly, there have to be factors that support a shared narrative; namely, the shared subject of discussion. We will refer to these as *semiotic* factors to emphasise that this kind of factor underwrites communication (Roy, 2005; Steels, 2011). In other words, semiotic factors entail latent states that exist only in the context of discourse; e.g., ‘we are discussing the *colour* of something’.

These four kinds of factors (*narrative*, *question*, *scenic* and *semiotic*) are sufficient to specify a question about something, or an answer, generated under beliefs about something. Crucially, some of these factors depend upon choices or *policies* and the others do not. For example, the agent can choose the form of a *question* and its *semiotic* content but cannot change *scenic* states (i.e., the scene or concept being discussed). In addition, we will assume that the narrative cannot be changed, in the sense that a question is always invited by a prompt and is followed by an answer. With this particular construction, agents can update their beliefs about *scenic* states on the basis of their beliefs about the current *semiotic* state and responses to questions. In other words, hidden states of the world can be communicated via shared semiotics that rest upon lawful answers to questions under a shared generative model. One can intuit that this generative model requires high-order interactions among the factors in play to generate sentences. In other words, the contingencies that generate a sequence of veridical questions and answers necessarily entail the interactions or conjunctions among several factors. Much of what follows is an attempt to illustrate these interactions using worked examples.

Having specified the form and semiotic content of a sentence, one can now generate a sequence of words in a subordinate level of a generative model that is equipped with probabilistic transitions among lexical states. The implicit transitions from word to word are prescribed by the narrative and question factors of the higher level to generate a *syntax*, while *semantic* content is specified by the semiotics. These two attributes (*syntactic* and *semantic*) constitute, in the example below, the hidden factors of the lower level of the model. Finally, given these two factors one can generate the appropriate *lexical* sequence of words; again, via an interaction of (*syntactic* and *semantic*) factors.

### 2.2 A generative model for “Twenty Questions”

**Fig. 2:**
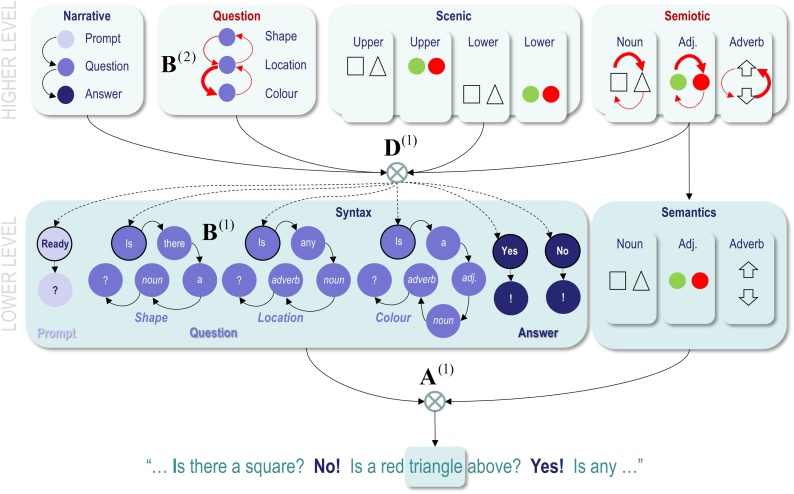
**A generative model for Twenty Questions**: This figure provides a schematic illustration of the generative model. This schematic displays the architecture involved in generating a sequence of words that could constitute a language-like narrative. In brief, this is a hierarchical (i.e., deep) generative model formulated in terms of discrete hidden states and outcomes (here, outcomes from the lower level are single words). The architecture is deep because there are two levels of hidden states, where the higher (deeper) level unfolds slowly in time—furnishing contextual constraints on the lower level that generates a sequence of words. The higher level contains hidden factors that generate the syntax and semantic content of a sentence, which are passed to the lower-level. Each panel uses a coloured shape to describe the different states of each factor. At the higher level, transitions among *narrative* states (**B**^(2)^) generate a sequence of phrases that cycle in a particular order through “Prompts”, “Questions” and “Answers”, where their form depends upon interactions with other hidden states in the generative model. The form of questions has been factorised into the type of *question* (“Shape”, “Location”, or “Colour”) and its *semiotic* content. The semiotic content has three factors (*noun*, *objective* and *adverb*), each with two states (*noun*: “square” or “triangle”; *adjective*: “green” or “red” and *adverb*: “below” or “above”). Similarly, the four *scenic* factors correspond to beliefs about the attributes of upper and lower objects in the world; namely, their *colours* (green or red) and *shapes* (square or triangle). In this generative model, choices about the type of question and its semiotic content are policy-dependent—as illustrated by the red arrows. In other words, policies determine transitions (encoded by the **B^(2)^** matrices) among controllable states, so that *question* and *semiotic* states are selected intentionally. For example, the question generated by the thick red arrows in the figure would be: “Is a red triangle above?” The combination of these states completely determines the *syntax* and *semantic* content of a sentence at the lower level (which is encoded in the matrix **D^(1)^)**. The hidden *syntax* states at the lower level comprise specific words, such as “Ready” and “Is”, grammar, such as “?” or “!”, and abstract representations, such as *noun*, *adverb*, and *adjective*. The words denoted by the abstract representations are determined by the *semantic* factor, which is isomorphic with the *semiotic* factor of the higher level. The first word of the phrase corresponds to the initial *syntactic* state at the lower level—which is determined by the interactions among states at the higher level, encoded by the mapping **D**. For example, if the narrative state is a *Question*, then the initial *syntax* state is the word “Is”, no matter which of the three *question* states are selected at the higher level. The B^(1)^ matrices then determine subsequent words (illustrated by the black arrows), by specifying transitions among *syntax* states that do depend upon the *question* states at the higher level. However, if the *narrative* state is *Answer*, then the initial *syntax* state can be “Yes” or “No”, depending upon high order interactions among the remaining high-level states: a “Yes” will be generated when, and only when, *scenic* and *semiotic* states are congruent (e.g., if the question “Is a red triangle above?” admitted a positive response, because a red triangle is in the upper location). For clarity, some syntaxes have been omitted; for example, a “Not sure” answer. In addition, this figure omits embellishments that generate synonymous phrases (e.g., “not sure”, “can't say”, and so on). The final stage is to map states at the lower level to outcomes at each time step of the generative process. This is denoted by the likelihood mapping **A**^(1)^. In the example highlighted here, the articulated word “triangle” depends upon the current syntax state being a noun and the associated content being a “triangle”. States without arrows are absorbing states; in other words, the state only transitions to itself.

Fig. 2 provides a schematic illustration of this kind of generative model and fills in some of the details (please see figure legend). This example will be used later to illustrate a simplified version of ‘Twenty Questions’, where a subject has to determine the configuration of two hidden objects by asking a series of closed questions in response to a prompt. The two objects are placed on top of each other and each object can either be a square or a triangle, which can be either red or green. This means that an ideal (active) Bayesian observer should be able to disclose the configuration with four questions: two questions to establish the colour and shape of the lower object and two questions do the same for the upper object. However, this depends upon *asking the right questions*, in relation to updated beliefs based upon previous answers. It is this epistemic, uncertainty reducing aspect of communicative exchange we hoped to demonstrate and characterise.

The particular levels of the factors in the generative model of Fig. 2 have been constructed to create a minimal model of how one agent can communicate beliefs about scenic states (i.e., configurations of hidden objects) to another. At the higher level, the model incorporates beliefs about the part of the *narrative* that is enacted (prompt, question, or answer), the type of *question* (shape, location, or colour), the putative *scenic* state of the world, and a *semiotic* factor indicating the topic of discussion. These factors generate expectations for the lower level: namely, the syntax (i.e., the ordering and content of words) and the semantics (i.e., which shapes, colours, and locations the agent is being questioned on). The lower level thus generates sequences of words, which are concatenated to form phrases—and sequences of phrases (i.e., exchanges) occur as the higher level cycles through the lower level.

Note that the ‘syntaxes’ included here would not all be considered as syntax under traditional definitions. In the current implementation, syntaxes are just sequences of states (words), with grammar used as terminating states to indicate that the conversational turn has ended.

The repertoire of syntactic structures within this model is limited to three sorts of questions and two sorts of answers; however, even with this limited repertoire, the combinatorics of what could be said is non-trivial. To ask a particular question, the subject has to choose the form of the question and the levels of the three semiotic factors by selecting the appropriate policy. To make sense of any answer, it also has to remember these choices. This memory is endowed by a higher level that maintains beliefs about controllable (i.e., question and semiotic) factors, after committing to a particular policy. The selected policy minimises uncertainty and will therefore change with beliefs about the hidden scene, over successive questions and answers, ensuring forward-looking exchanges (Allwood et al., 1992). Note that this kind of working memory—and epistemic behaviour—emerges naturally from maximising model evidence (i.e., minimising variational free energy), given a generative model of successive states of the world that generate outcomes.

We included a prompt state simply to demonstrate the cycling between prompts, questions, and answers. In this formulation, the prompt conveys no interesting information: it is merely part of the structured dialogue. In the following simulations, we simply use it to convey the type of turn-taking that is often seen in realistic settings.

Clearly, there are many ways in which we could carve up the factors or causes that underwrite linguistic exchanges, and we have ignored many interesting aspects; however, the basic message is that one needs to consider the factorisation and the deep (hierarchical) nature of generative models before dissecting the computational architecture of language. In what follows, we consider more broadly how generative models of this sort can be represented in graphical form, and how variational message passing generates predictions for neuronal dynamics.

## 3 Active inference

The previous section considered the form of a generative model. We can now use active inference to simulate action and perception under that model. The procedures used here assume that the brain restricts itself to a limited number of characteristic states (Friston, 2013)—a property that all sentient systems must possess. Mathematically, these procedures minimise surprise (in information theoretic terms), which is equivalent to maximising Bayesian model evidence; in other words, they maximise the probability of sensory exchanges with the environment, under a generative model of how those sensations were caused. This is the essence of active inference, and implicit self-evidencing (Hohwy, 2016).

Intuitively, self-evidencing means the brain can be described as inferring the causes of sensory samples while, at the same time, soliciting sensations that are the least surprising (e.g., not looking at the sun directly or maintaining thermoreceptor firing within a physiological range). Technically, this take on action and perception can be cast as minimising a proxy for surprise, known as variational free energy. From a statistical perspective, variational free energy can be decomposed into *complexity* and *accuracy*, such that minimising variational free energy provides an accurate account of some data in the simplest way possible (Maisto et al., 2015). Crucially, active inference generalises Bayesian inference, such that the objective is not just to infer the latent or hidden states that cause sensations but to act in a way that minimises expected surprise. In information theory, expected surprise is known as entropy or uncertainty. This means, one can define optimal behaviour as acting to resolve uncertainty: e.g., saccading to salient, or information rich, regimes of visual space or avoiding outcomes that are, *a priori*, costly or unattractive. In the same way that direct action and perception minimise free energy, action can be specified in terms of plans or policies that minimise the free energy expected on pursuing that policy.

This section briefly reviews parts of active inference that are relevant to the current paper. We begin by explaining expected free energy. We then consider how active inference is applied to discrete generative models, such as the model described in the previous section. Finally, we consider how belief updating can be implemented as a neuronally plausible message passing scheme.

### 3.1 Expected free energy

Expected free energy (*G*) has a relatively simple form (see Appendix A), which can be decomposed into an epistemic, information seeking, uncertainty reducing part (*intrinsic value*) and a pragmatic, goal seeking, cost aversive part (*extrinsic value*). Formally, the expected free energy for a particular policy (π) can be expressed in terms of posterior beliefs [$Q(o_{\tau },s_{\tau })=P(o_{\tau }|s_{\tau })Q(s_{\tau }|\pi )$] about outcomes (*o*) and states (*s*) of the world at time τ in the future:

(1) $$G(\pi ,\tau )\geq -\underset{\text{intrinsic value}}{\underset{︸}{E_{Q}[\ln Q(s_{\tau }|o_{\tau },\pi )-\ln Q(s_{\tau }|\pi )]}}-\underset{\text{extrinsic value}}{\underset{︸}{E_{Q}[\ln P(o_{\tau })]}}$$

Extrinsic (i.e., pragmatic) value is simply the expected value of a policy defined in terms of outcomes that are preferred *a priori*; where the equivalent cost corresponds to Bayesian risk or prior surprise (see Table 1 and Appendix B). The more interesting part is the uncertainty resolving or intrinsic (i.e., epistemic) value, variously referred to as relative entropy, mutual information, information gain, Bayesian surprise or the value of information expected under a particular policy (Barlow, 1961; Howard, 1966; Itti and Baldi, 2009; Linsker, 1990; Optican and Richmond, 1987). An alternative formulation of expected free energy can be found in Appendix A: this formulation rearranges the equation for expected free energy, so that it is cast as the expected uncertainty about outcomes (i.e. *ambiguity* or expected *inaccuracy*) plus the Kullback-Leibler divergence between predicted and preferred outcomes (i.e., *risk* or expected *complexity*). This formulation shows that minimising expected free energy is guaranteed to realise preferred outcomes, while resolving uncertainty about the states of the world generating those outcomes.

**Table 1:** Expressions pertaining to models of discrete states: the shaded rows describe hidden states and auxiliary variables, while the remaining rows describe model parameters and functionals.

Expression	Description
$\begin{array}{c} o_{\tau }\in \{0,1\}\\ o_{\tau }=\sum_{\pi }\pi_{\pi }\cdot o_{\pi ,\tau }\in [0,1] \end{array}$	Outcomes and their posterior expectations
$\begin{array}{c} s_{\tau }\in \{0,1\}\\ s_{\tau }=\sum_{\pi }\pi_{\pi }\cdot s_{\pi ,\tau }\in [0,1] \end{array}$	Hidden states and their posterior expectations
$o_{\pi ,\tau }=As_{\pi ,\tau }$	Expected outcome, under a particular policy
$\begin{array}{c} \pi \in \{1,\ldots ,K\}\\ \pi =(\pi_{1},\ldots ,\pi_{K})\in [0,1] \end{array}$	Policies specifying state transitions and their posterior expectations
$\begin{array}{c} \nu_{\pi ,\tau }=\ln s_{\pi ,\tau }:\varepsilon_{\pi ,\tau }={\dot{v}}_{\pi ,\tau }\\ s_{\pi ,\tau }=\sigma (\nu_{\pi ,\tau }) \end{array}$	Auxiliary variable representing depolarisation and expected state, under a particular policy
$\begin{array}{c} \varepsilon_{\pi ,\tau }=\ln B_{\pi ,\tau -1}s_{\pi ,\tau -1}+\ln B_{\pi ,\tau }\cdot s_{\pi ,\tau +1}\\ +\ln A\cdot o_{\tau }-\ln s_{\pi ,\tau } \end{array}$	Auxiliary variables representing state prediction error
A	The likelihood of an outcome under each hidden state
$B_{\pi ,\tau }$	Time dependent probability transition matrices specified by the policy
$C_{\tau }=-\ln P(o_{\tau }|\pi )$	Prior surprise about outcomes; i.e. prior cost or inverse preference
D	(Empirical) Prior expectations about initial hidden states
$F_{\pi }=\sum_{\tau }F(\pi ,\tau )$	Variational free energy for each policy
$G_{\pi }=\sum_{\tau }G(\pi ,\tau )$	Expected free energy for each policy
$G_{o}=G(o)$	Expected free energy for next outcome
$\sigma {\left(-G\right)}_{\pi }=\frac{\exp(-G_{\pi })}{\sum_{\pi }\exp(-G_{\pi })}$	Softmax function, returning a vector that constitutes a proper probability distribution.

Here, we are less concerned with the pragmatic aspect of expected free energy and focus on the epistemic drive to reduce uncertainty. We have previously addressed this epistemic affordance in terms of saccadic eye movements—to provide a constructivist explanation for visual searches: c.f., scene construction (Hassabis and Maguire, 2007; Mirza et al., 2016). In this paper, we use a more sophisticated generative model to illustrate the same sort of epistemic foraging, mediated by linguistic exchange. It is worth bearing in mind that the purposeful, inquisitive and abductive behaviours we will see later are all emergent properties of minimising (expected) free energy. In other words, there is no need to handcraft any rules or grammar, or provide any reinforcement or feedback. All of the behaviours shown in this paper result from the structure of the generative model. Subsequent sections will illustrate the belief updating under this model—and so, first, we consider how belief updating relates to neuronal processes and action.

### 3.2 Belief updating and neuronal dynamics in discrete generative models

This section focuses on generative models of discrete outcomes caused by discrete states that cannot be observed directly (i.e., hidden states). This summary is based on (Friston et al., 2017c), which contains more detail for readers who are interested in the approach. In brief, the unknown variables in these models correspond to *states* of the world—that generate outcomes—and the *policies* that generate successive states. For simplicity, we introduce belief updating in terms of a generic discrete generative model, which has a single level; we then extend this description to discrete hierarchical models containing two levels.

**Fig. 3:**
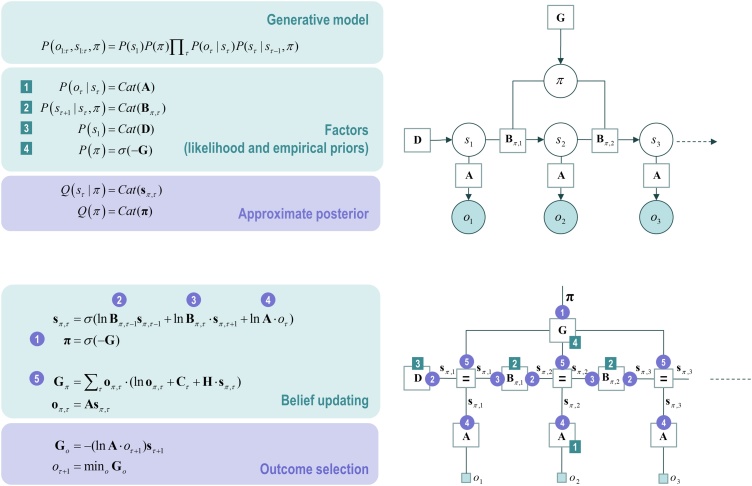
**Generative models for discrete states and outcomes. Upper left panel**: These equations specify the generative model. A generative model is the joint probability of outcomes and their (latent or hidden) causes, see first equation. Usually, the model is expressed in terms of a *likelihood* (the probability of consequences given causes) and *priors* over causes. When a prior depends upon a random variable it is called an *empirical prior*. Here, the likelihood is specified by a matrix **A**, whose elements are the probability of an outcome under every combination of hidden states. The empirical priors pertain to probabilistic transitions (in the **B** matrix**)** among hidden states that can depend upon action, which is determined probabilistically by policies (sequences of actions encoded by *π*). The key aspect of this generative model is that policies are more probable *a priori* if they minimise expected free energy **G,** which depends upon prior preferences about outcomes or *costs* encoded by **C**. Finally, the vector **D** specifies the initial state. This completes the specification of the model in terms of its parameters; namely, **A**, **B**, **C** and **D**. Bayesian model inversion refers to the inverse mapping from outcomes to causes; i.e., estimating the hidden states that cause outcomes. In approximate Bayesian inference, one specifies the form of an approximate posterior distribution. This particular form in this paper uses a mean field approximation, in which posterior beliefs are approximated by the product of marginal distributions over time points. Subscripts index time (or policy). See Section 2 and Table 1 for a detailed explanation of the variables (italic variables represent hidden states, while bold variables indicate expectations about those states). **Upper right panel**: This Bayesian network represents the conditional dependencies among hidden states and how they cause outcomes. Open circles are random variables (hidden states and policies) while filled circles denote observable outcomes. Squares indicate fixed or known quantities, such as the model parameters. **Lower left panel**: these equalities are the belief updates mediating approximate Bayesian inference and outcome selection. When the agent is responsible for generating outcomes (e.g., speaking), they are selected to minimise free energy or, in other words, maximise accuracy under posterior beliefs about the next state of the world. **Lower right panel**: this is an equivalent representation of the Bayesian network in terms of a Forney or normal style factor graph. Here the nodes (square boxes) correspond to factors and the edges are associated with unknown variables. Filled squares denote observable outcomes. The edges are labelled in terms of the sufficient statistics of their marginal posterior. Factors have been labelled in terms of the parameters encoding the associated probability distributions (on the upper left). The circled numbers correspond to the messages that are passed from nodes to edges (the labels are placed on the edge that carries the message from each node). The key aspect of this graph is that it discloses the messages that contribute to the posterior marginal over hidden states; here, conditioned on each policy. These constitute [*forward*: ❷] messages from representations of the past, [*backward*: ❸] messages from the future and [*likelihood*: ❹] messages from the outcome. Crucially, the past and future are represented at all times so that as new outcomes become available, with the passage of time, more likelihood messages participate in the message passing; thereby providing more informed (approximate) posteriors. This effectively performs online data assimilation (mediated by forwarding messages) that is informed by prior beliefs concerning future outcomes (mediated by backward messages). Please see Table 1 for a definition of the variables in this figure. Adapted with permission from (Friston et al., 2017c).

Fig. 3 describes the basic form of these generative models in two complementary formats, and the implicit belief updating following the observation of new (sensory) outcomes. The equations on the left specify the generative model in terms of a probability distribution over outcomes, states and policies that can be expressed in terms of marginal densities or factors. These factors are conditional distributions that entail conditional dependencies—encoded by the edges in the Bayesian network on the upper right. The model in Fig. 3 generates outcomes in the following way. First, a policy (i.e., a plan or controlled action sequence) is selected at the highest level using a softmax function of the free energy expected under plausible policies. Sequences of hidden states are then generated using the probability transitions specified by the selected policy (encoded in **B** matrices). As the policy unfolds, the states generate probabilistic outcomes at each point in time (encoded in **A** matrices).

The equivalent representation of this graphical model is shown as a Forney factor graph on the lower right. Here, the factors of the generative model (numbers in square boxes) now constitute the nodes and the (probability distribution over the) unknown states are associated with edges. The rules used to construct a factor graph are simple: the edge associated with each variable is connected to the factors in which it participates.

After specifying the generative model, we can use standard belief updating schemes (Friston et al., 2017c) that have been used in previous applications of active inference (e.g., Adams et al., 2013; Mirza et al., 2016). These message passing schemes are neuronally plausible, and minimise free energy. In brief, the average transmembrane potential of a neuronal population is assumed to reflect the logarithm of an expected hidden state, under a particular policy: $\nu_{\pi ,\tau }=\ln s_{\pi ,\tau }$. By introducing an auxiliary variable (i.e., state prediction error), one obtains the following update scheme, whose solution satisfies the belief update equations in the lower left panel of Fig. 3.

(2) $$\begin{array}{c} \varepsilon_{\pi ,\tau }=\ln B_{\pi ,\tau -1}s_{\pi ,\tau -1}+\ln B_{\pi ,\tau }\cdot s_{\pi ,\tau +1}+\ln A\cdot o_{\tau }-\ln s_{\pi ,\tau }\\ {\dot{v}}_{\pi ,\tau }=\varepsilon_{\pi ,\tau }\\ s_{\pi ,\tau }=\sigma (v_{\pi ,\tau }) \end{array}$$

Although we employ a marginal message passing scheme in the simulations presented later, the derivations presented here use a mean-field approximation to simplify the expressions. While we could have used a mean field approximation and the ensuing variational message passing, this tends to lead to overconfident inferences. Practically, there is little difference between the two (Parr et al., 2019a): both rely upon the synthesis of local messages from the Markov blankets of variables in the factor graph.

These differential equations correspond to a gradient descent on variational free energy as described in (Friston et al., 2017a) and Appendix B:

(3) $$\begin{array}{c} {\dot{v}}_{\pi ,\tau }=\varepsilon_{\pi ,\tau }=-\frac{\partial F_{\pi }}{\partial s_{\pi ,\tau }}\\ F_{\pi }=\sum_{\tau }F(\pi ,\tau )\\ F(\pi ,\tau )=\underset{\text{complexity}}{\underset{︸}{E_{Q(s_{\tau -1}|\pi )}[D[Q(s_{\tau }|\pi )||P(s_{\tau }|s_{\tau -1},\pi )]]}}-\underset{\text{accuracy}}{\underset{︸}{E[\ln P(o_{\tau }|s_{\tau })]}} \end{array}$$

Crucially, in terms of neuronal dynamics, the sigmoid function (*σ*) in Eq. 2 can be thought of as a sigmoid (firing rate) activation function of transmembrane potential, and log expectations about hidden states can be associated with depolarisation of neuronal populations encoding expectations. This has some construct validity in relation to theoretical proposals and empirical work on evidence accumulation (de Lafuente et al., 2015; Kira et al., 2015) and the neuronal encoding of probabilities (Deneve, 2008). Equivalent updates can be derived for beliefs about policies and the precision of those beliefs. Although omitted from Fig. 3 for simplicity, the expected precision of beliefs about policies is interesting because it has all the hallmarks of phasic dopamine dynamics. We will look briefly at simulated dopaminergic firing later. Interested readers are referred to (Friston et al., 2017a, 2014) for details.

As noted above, in this (pure communication) setting, outcomes are generated by the agent who is currently speaking. These outcomes are those that minimise variational free energy. As can be deduced from Eq. 3, these are simply the outcomes that maximise accuracy:

(4) $$\begin{array}{c} o_{\tau +1}=\min_{o}G_{o}\\ G_{o}=-E_{Q}[\ln P(o_{\tau +1}|s_{\tau +1})]\\ =-(\ln A\cdot o_{\tau +1})s_{\tau +1} \end{array}$$

This follows from the fact that the complexity part of free energy does not depend upon outcomes (see Eq. 3). This sort of outcome is formally related to motor output under active inference; namely, the fulfilment of proprioceptive predictions by classical reflexes (Adams et al., 2013; Shipp et al., 2013). In the current simulations, words or phrases are generated, which play the equivalent role of fulfilling predictions based upon beliefs about hidden states at each point in time.

**Fig. 4:**
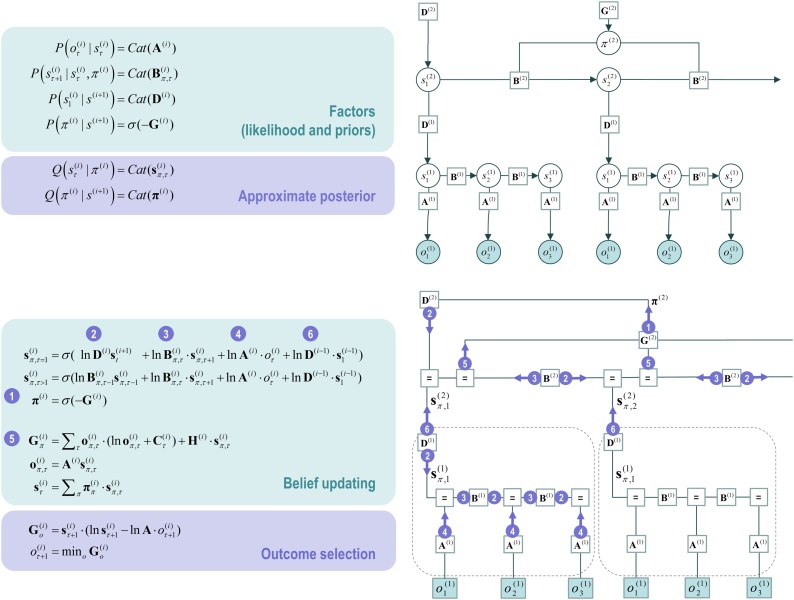
**Deep temporal models. Left panel**: This figure provides the Bayesian network and associated Forney factor graph for deep temporal models, described in terms of factors and belief updates on the left. The graphs adopt the same format as Fig. 3; however, here the model has been extended hierarchically, where (bracketed) superscripts index the hierarchical level. The key aspect of this model is its hierarchical structure that represents sequences of hidden states over time or epochs. In this model, hidden states at higher levels generate the initial states for lower levels, which unfold to generate a sequence of outcomes: c.f., associative chaining (Page and Norris, 1998). Crucially, lower levels cycle over a sequence for each transition of the level above. This is indicated by the subgraphs enclosed in dashed boxes, which are ‘reused’ as higher levels unfold. It is this scheduling that endows the model with deep temporal structure. The probability distribution over initial states is now conditioned on the state (at the current time) of the level above. Practically, this means that **D** now becomes a tensor, as opposed to a vector. The messages passed from the corresponding factor node rest on Bayesian model averages that require the expected policies [*message* ❶] and expected states under each policy. The resulting averages are then used to compose descending [*message* ❷] and ascending messages [*message* ❻] that mediate the exchange of empirical priors and posteriors between levels, respectively. Adapted with permission from (Friston et al., 2017c).

The final step is to create deep generative models by stacking generative models on top of each other; such that the outcomes generated by one level provide (empirical) priors on the initial states of the level below. By linking hierarchical levels in this fashion, states at the higher level change slowly over time, because states higher level remain the same throughout a sequence of state transitions at the lower level. In the current setting, this means that beliefs about successive words at the lower level are updated on a faster timescale than beliefs about a phrase at the higher level—obliging a phrase to consist of multiple words. Top-down (empirical) priors from the higher level provide a context for inference about the next word, which is informed by all the preceding words in a sentence. This is an important aspect of deep temporal models that lends inference a hierarchical nature; known technically as a semi-Markovian process. Fig. 4 illustrates the hierarchical form of the generative model (upper panels) and the accompanying message passing scheme (lower panels) in the form of a factor graph. Note that Fig. 4 is simply an extension of Fig. 3. At the higher level, the likelihood mapping from hidden states to outcomes (**A**) from Fig. 3 is replaced by a mapping from hidden states in the higher level to the initial states of a lower-level (denoted by **D**). These mappings allow interactions between states at the higher level to influence states in the lower level.

**Fig. 5:**
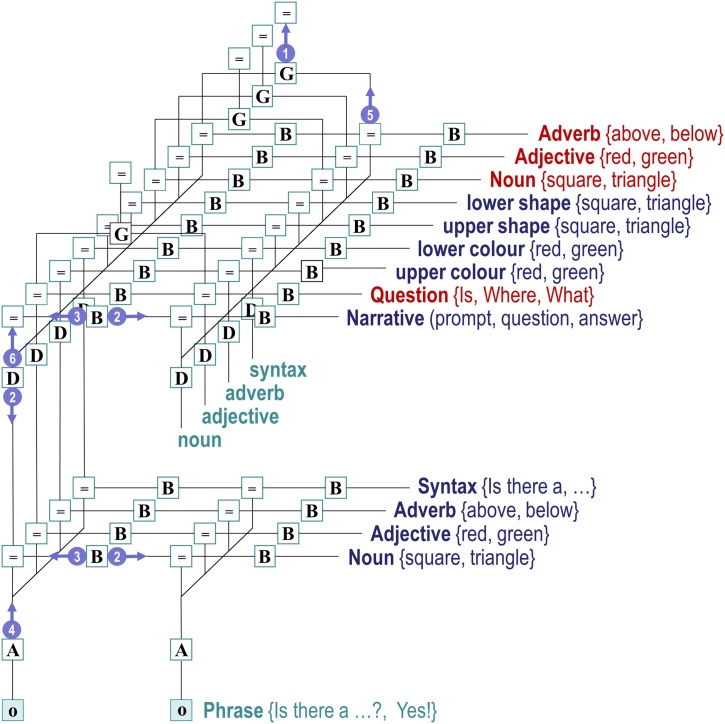
**Factor graph for 20 questions:** this schematic illustrates the message passing using a Forney style factor graph for the generative model in Fig. 2, using the format of Fig. 4. In this schematic, we have unpacked the hidden state factors, labelling those with multiple (policy-dependent) probability transition matrices in red. This graphic was produced automatically using the SPM software (please see software note).

In the spirit of Fig. 4, Fig. 5 shows the particular Forney style factor graph for the generative model of Twenty Questions in Fig. 2. Here, the hidden states have been unpacked into their factors, where controllable states are labelled in red. As noted above, controllable states have transition probabilities that are prescribed by a policy. For example, for the semiotic factor *noun*, the available policies could move the semiotic state ‘square’ to ‘triangle’. Here, the policy remains in play for a succession of state transitions. In other words, once a policy is inferred or selected, it remains operational in terms of predicting successive outcomes. For example, if I commit to the semiotic state ‘square’, then it remains the subject of the next question *and subsequent answer*. Recall that policies are selected to minimise expected free energy (Section 3.1).

Equipped with this model and variational message passing scheme, we are now in a position to simulate conversations; both in terms of belief updating and associated neuronal message passing. When the agent is listening, the outcomes can be generated by another agent to simulate dyadic exchange. Conversely, when the agent is talking, outcomes are selected to minimise the free energy under the agent’s beliefs. In other words, when the agent is talking, it selects the least surprising words, given its beliefs about the current syntax and semantics. Notice that the agent does not ‘know’ who is talking—it just expects to hear things that are consistent with its beliefs. If it hears something that is surprising or unexpected, the agent will update its beliefs about the scene and semiotics currently in play. More importantly, the agent’s beliefs about what is being said depend upon the policies inferred. These policies minimise expected free energy, which means the agent expects to encounter salient, informative answers and, crucially, questions. In other words, it expects to hear questions and answers that resolve uncertainty, which will be the same as the questions it would ask and the answers it would supply.

When considering hierarchical generative models of language processing, we are confronted with the linearization problem (Bornkessel et al., 2005): namely, how are outcomes supplied to higher levels of the generative model and, how do higher levels provide constraints on evidence gathering at lower levels? In other words, how can one accumulate evidence from sequential stimuli to form beliefs about things that do not change with time? Happily, this problem that has already been solved by deep temporal models of the sort above. We demonstrate the implicit message passing and belief updating that underwrites this form of (linearised) evidence accumulation in the next section, by simulating an agent playing the “Twenty Questions” game.

## 4 “Twenty questions” simulations

To illustrate belief updating—and its neuronal correlates—we use a simplified version of “Twenty Questions”. Specifically, we simulated conversations comprising six *exchanges*, where each exchange comprises three *phrases* or sentences. The phrases always followed the same sequence: a prompt, a question, then an answer. This order was fixed by specifying very precise priors about transitions among *narrative* states. Each phrase comprised up to six words, and each word was processed with belief updates described by Eq. 3. These updates were evaluated in 16 time-steps of 16 ms (of simulated and approximately real time). This meant that words were generated every 256 ms, such that a sentence of four words takes about a second to articulate. In these simulations, the artificial agent could take the role of the questioner or the answerer: the agent either listened for the prompt, asked a question, and then listened to the answer, or issued the prompt, listened for a question, then supplied the answer. In all cases, the agent (slightly) preferred affirmative answers (“Yes”) over negative answers (“No”). These preferences were specified by setting prior costs of **C** = –¼ for “Yes” and **C** = ¼ for “No” (see Table 1). This means that the agent will ask questions that it believes will elicit a “Yes” answer, everything else being equal.

In these simulations, the agent started out with uniform prior beliefs about which colour and shape was present at the two locations (above and below). It played the role of the questioner for the first four exchanges, after which it identified the colours and shapes of both objects with high confidence. Having updated its beliefs, it then switched roles to answer two questions. To allow the agent to play the roles of the questioner and answerer for these simulations, we separated the agent’s generative model from the generative process; effectively, this means that the agent was ‘in conversation’ with the generative process. The generative process had exactly the same form as the generative model, except the generative process had more precise beliefs about the scene. Here, as the agent was not equipped with beliefs about whether it was speaking or listening, we simulated turn-taking by sampling the output from the agent or generative process at the appropriate stages of the exchange.

**Fig. 6:**
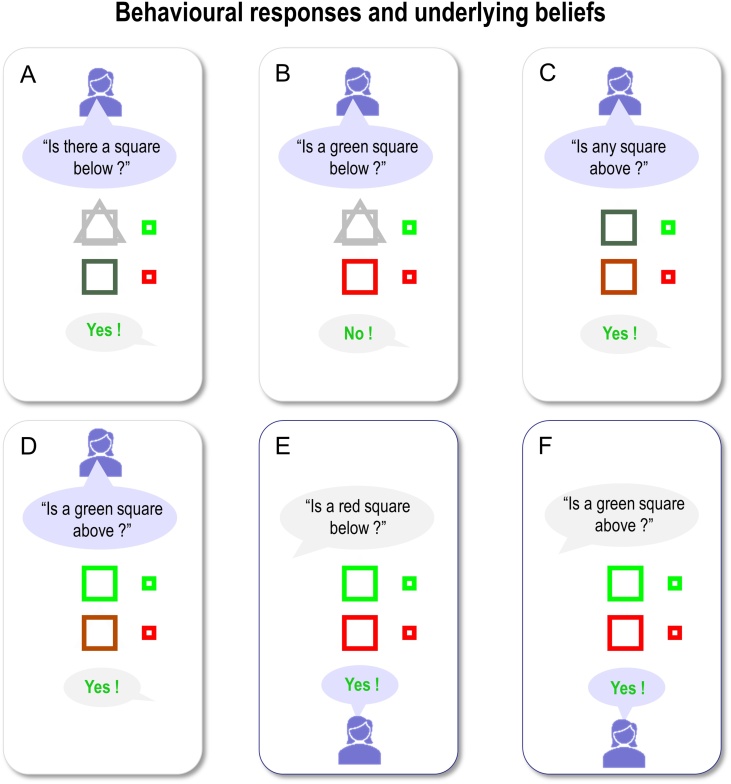
**Behavioural responses**: Each panel shows the posterior expectations of a synthetic subject after its question had been answered. The agent’s beliefs about *shape* (square versus triangle) and *colour* (green versus red) for the upper and lower locations are depicted with large icons. Where the agent has no particular (i.e., uniform) beliefs, the two shapes are displayed overlaid and/or in grey (e.g., upper locations in panels A and B); where the agent’s beliefs tend toward a particular colour, the shape is shaded slightly red or green. The true scene (with veridical colours and shapes) is shown with small icons to the right. The question is shown in black text (above each set of expectations), while the answer is shown below. All of the answers in this simulation are correct, so they are displayed in green text. The human icons and purple callouts are positioned next to the agent’s vocalisations, to illustrate whether the subject was asking questions (first four exchanges) or answering them (last two exchanges).

Fig. 6 displays the results of belief updating. Each panel shows posterior expectations about the two hidden objects after each of the six questions were answered. The questions are shown in black text towards the top of the panel, while the answer is shown at the bottom of the panel. All answers in this example were correct (i.e., correspond to the true scene), so are displayed in green. Within each panel, the agent’s beliefs about *shape* (square versus triangle) and *colour* (green versus red) at the two locations are depicted with large icons. The true (narrative) scene is shown with small icons to the right.

During the first four questions, the agent accumulates evidence and builds veridical beliefs about the scene at hand. At the beginning, it has no particular (i.e. uniform) beliefs about the shapes and colours at the two locations. First, it chooses to ask a question about the shape, because it is more likely to get an affirmative (preferred) response than if it were to ask a question about shape and colour together. After the first answer, it knows there is a square below (see first panel) and subsequently asks a question about the combination of shape and colour. After the second answer (see second panel), it knows that the square is not green and must therefore be red. It then goes on to ask similar questions for the upper location, after which time it holds precise beliefs about the shapes and colours at the two locations. By the time it answers the fifth and sixth questions, the agent can provide veridical answers to questions about specific scene components (the fact these responses are in green text indicates that the answers are correct).

Notice that the expectations of the colour (red) of the lower panel become less precise after first inferring there is a red square below (compare second and third panels). This arises because we have slowed down belief updating, so that its time constants correspond roughly to those observed empirically (see below). This precludes complete convergence to free energy minima during belief updating. The ensuing uncertainty is then carried over to the next exchange. Further, notice that after responding correctly to the question about the colour of the lower square (fifth question; lower middle panel), the agent’s beliefs are refreshed as the answer provides confirmatory evidence about what was believed.

As anticipated, the artificial agent resolved uncertainty about the hidden scene after only four questions, suggesting that appropriate questions were asked. For example, the first question establishes that there is a square below, while the second discloses the fact that it is red. It could have opted to ask only *What* questions, but then it could have needed as many as 8 questions to infer the correct scene. Notice also that the second question is not redundant: it is asked in the context of knowledge that the lower object is square. A possible second question would have been to ask: “Is there a circle below?”, but given (i) the agent already knows the lower object is a square and (ii) in this scenario only one object is present at each location, this question would not reduce uncertainty about the contents of the scene. Ultimately, the behaviour demonstrated in these simulations emerges because the agent selected policies that reduced uncertainty about the scene. This can only happen because the generative model entertains future states, which enable the agent to evaluate expected outcomes in the future. For example, any answer to the second question (“Is a red square below”) completely resolves uncertainty about its colour. The agent knows this question will resolve uncertainty before the question is even asked. Thus, this type of question has epistemic value.

Note the subtle nature of this epistemic behaviour: the agent is using semiotics states (*noun*, *adjective*, and *adverb*), over which it (believes it) has control, to resolve uncertainty about *scenic* states, over which it (believes it) has no control. In this scenario, the agent exerts control by generating outcomes (e.g., questions); it will generate outcomes that are the least surprising, under uncertainty resolving policies. This vicarious belief updating is central to the current formulation when considering how we might install beliefs in others through linguistic communication.

### 4.1 Message passing and neurophysiology

**Fig. 7:**
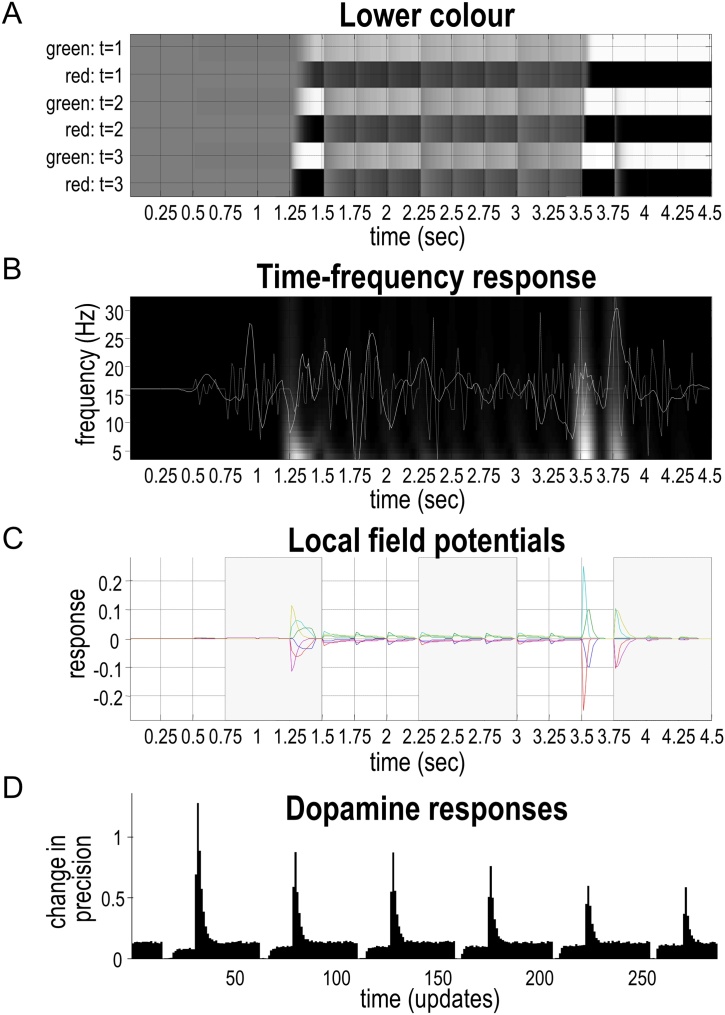
**Electrophysiological responses**: This figure shows the simulated electrophysiological responses associated with the belief updating reported in Fig. 6. In this figure, we focus on beliefs about the *colour* of the lower object, which is at the higher level of the generative model—thus, these plots show simulated responses following each phrase (i.e., prompt, question, and answer) rather than following each word. The horizontal axes show time over the entire exchange, assuming each phrase lasts for 250 ms. Expectations about the hidden state encoding the *colour* of the lower object are presented in raster format in the panel A, where black corresponds to maximum firing rates. Panel B shows the same data but in a different format: here, pooled expectations (filtered between 4 and 32 Hz) are shown as a white line. This simulated local field potential is superimposed upon a time-frequency heat map to illustrate bursts of frequency-specific energy (white), during periods of belief updating. The underlying fluctuations in simulated neuronal activity, after bandpass filtering between 4 Hz and 32 Hz, are shown in panel C. Each of the coloured lines on this plot represent belief updating for a given unit (i.e., the rows of the upper panel). Panel D shows simulated dopamine responses after each answer: these attenuate as uncertainty is resolved.

Having illustrated belief updating behaviour, we now take a closer look at the predictions of this type of inference, or sequential evidence accumulation, for neurophysiology. Fig. 7 illustrates electrophysiological and dopaminergic responses to the six questions from the simulation above. These responses are shown in various formats: Fig. 7A shows posterior expectations about the colour of the lower object at various times during the sequence of six narratives, displayed in raster plot format. There are two temporal scales of belief updating: the convergence to minimum free energy following each new stimulus, and faster dynamics that underwrite that convergence. Usually, new stimuli are assumed to be sampled every 250 ms. This period allows 16 rounds of variational message passing to converge to a free energy minimum, where each round or iteration is considered to last about 16 ms. These assumptions render synthetic neuronal responses consistent with the time constants of ERPs in the brain(Friston et al., 2017a, d). For simplicity, this figure displays only the *colour* states. Different units are labelled on the Y-axis; namely, green or red at successive epochs (1, 2, and 3) within each exchange.

During the time window corresponding to the first question and answer (0–0.75 s), Fig. 7A is shaded in grey, indicating that the agent has uniform beliefs about the colour of the object during the first question, which queries the shape and not colour. The second question asks about the colour of the lower object. This question (Fig. 6B) has the answer “No” (i.e., not green), indicating that the colour of the square at the lower position must be red. The plot shows that this answer induces profound belief updating; the belief that the lower square is red is very precise and this belief is maintained (i.e., ‘remembered’) throughout the exchange (i.e., for the remainder of the time plotted). During this time, the shading on this plot allows us to visualise the reduction in precision for the belief that the object is red, and subsequent reinstatement of precision after the fifth question—as discussed in the previous section.

Notice in Fig. 7A that the latency of this belief updating for expectations at the beginning of the trial is greater than at the end—this is due to message-passing backwards in time (message ❸ in Fig. 4). As noted above, these posterior beliefs decay a little over subsequent trials, until the agent reaffirms its conviction that the lower colour is indeed red.

Fig. 7B shows the same data in a different format. Here, pooled expectations (after filtering between 4 and 32 Hz) are shown as a white line. This is superimposed upon a time-frequency heat map to illustrate bursts of frequency-specific energy during periods of belief updating. We will examine this characterisation in more detail later, with reference to the next figure.

Fig. 7C illustrates the simulated fluctuations in neuronal activity, after bandpass filtering. These can be regarded as simulated local field potentials or event related potentials (Leonard et al., 2016), corresponding roughly to the voltage fluctuations in Eq. 3. Later in the paper, we will revisit these synthetic ERPs to characterise responses to surprising outcomes. The current simulation simply shows that the amplitude of simulated ERPs is related to the amount of information conveyed by an outcome—it shows greater responses to more informative parts of the narrative.

Finally, Fig. 7D shows simulated dopamine responses (i.e., expected precision of beliefs about policies), as described in (Friston et al., 2014). Interestingly, the peaks of these phasic responses coincide with times that answers are given. The key point to take from these phasic responses is that the implicit changes in confidence—about the policies being pursued—depends on the extent to which answers resolve uncertainty and fulfil prior preferences. Every time the agent receives (and to a lesser extent delivers) an answer, it becomes more confident about what it is doing. However, becoming more confident about the hidden scene attenuates the ‘confidence boosts’ (i.e., phasic dopamine responses). Anecdotally, this seems consistent with the subjective experience of “Twenty Questions”, where each confirmatory answer is rewarding, especially at the beginning of the game.

**Fig. 8:**
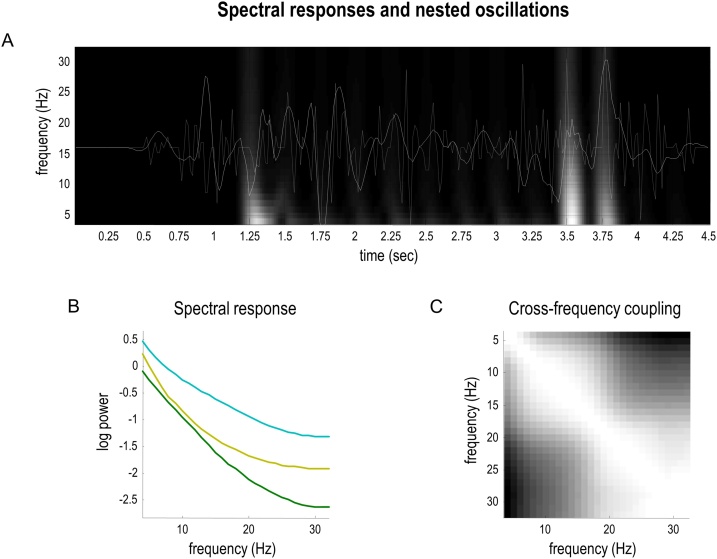
**Spectral responses and nested oscillations**. This figure shows the spectral responses associated with the simulated electrophysiological responses in Fig. 7. Panel A is a reproduction of Fig. 7B. Panel B reports the spectral density of the six units (i.e., ‘red’ or ‘green’ for epochs 1, 2, and 3). Only three lines are visible because pairs of responses overlap perfectly. Note that the scale is expressed in terms of log power. The matrix in panel C shows the correlation between the magnitudes of responses over frequencies ranging from 4 to 32 Hz. These correlations are based on the time frequency response in panel A.

Fig. 8 presents the simulated electrophysiological responses from Fig. 7 in terms of what one would predict when analysing spectral responses from the higher order area during belief updating. The lower panels show the spectral responses. Fig. 8B reports the log spectral density of the six units (i.e., neuronal populations), whose event related responses are shown in Fig. 7C. This shows that spectral responses show a degree of scale-free broadband activity, reflecting the fact that the simulated neuronal dynamics have multiple nested timescales.

The ensuing nonlinear coupling between fluctuations at different frequencies is summarised in terms of cross frequency coupling in Fig. 8C. This simple characterisation is the correlation between the response magnitudes, over frequencies ranging from 4 to 32 Hz (based on the time frequency response in the lower panel). The key thing to note from this panel is the off-diagonal structure: the lighter shading in the lower left and upper right quadrants of the plot indicate above-zero correlations, suggesting that there are correlations among the lower and higher frequencies—in other words, amplitude-to-amplitude coupling between theta and gamma responses. This coupling arises because belief updates at different temporal scales are likely to co-occur (i.e., at the same times) under this hierarchical model.

The belief updates under this hierarchical model also necessitate responses that would be interpreted as reflecting phase-amplitude coupling. These types of responses arise from belief updating at different hierarchical levels of the generative model, which occur at different temporal scales. Neuronal dynamics perform a gradient descent on variational free energy, as each new outcome becomes available (for the higher level, when each phrase is spoken). By virtue of this temporal scheduling, there are necessarily nested oscillations in the sense that fast (e.g., gamma) fluctuations unfold at a slow (e.g., theta) rhythm (Friston et al., 2017a): a succession of transients containing high-frequency components is induced by hearing each word, and these transients recur at the lower frequency of word presentation. In this class of hierarchical generative model, each transition at the higher level is accompanied by a ‘resetting’ of states at the lower level (Friston et al., 2017c). In the current application, phrase-level inferences generate the words contained within the phrase, and then the lower level ‘resets’ for the next phrase. This nesting naturally leads to phase-amplitude coupling—which is the most commonly studied type of cross frequency coupling (Canolty and Knight, 2010).

**Fig. 9:**
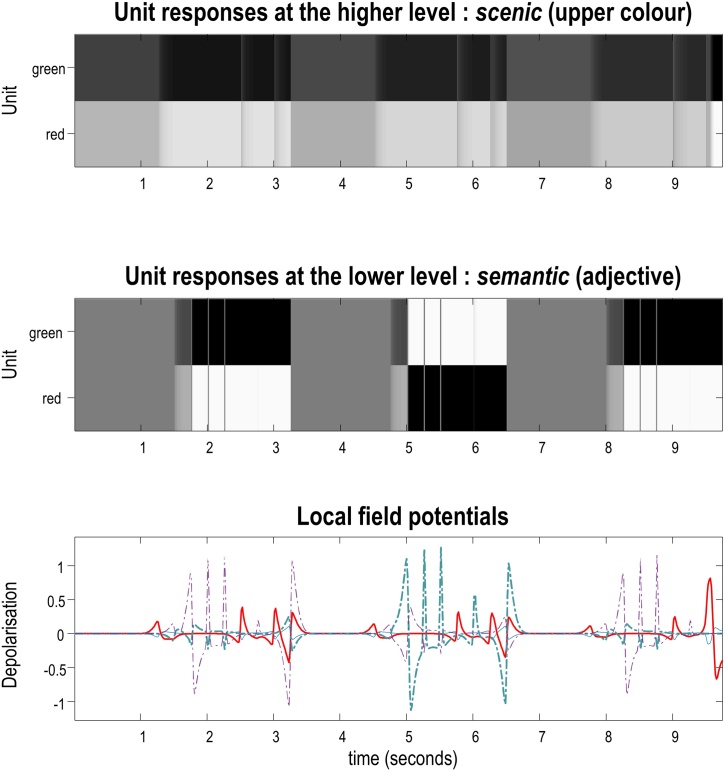
**Hierarchical message passing and nested oscillations**. The upper panel illustrates responses at the second level using the format of the upper panel of Fig. 7. Here, we focus on representations of the colour of the upper object—following each phrase—for the last three exchanges. At this point, the agent is fairly sure the upper object is green (as indicated by the darker shading for the ‘green’ unit in the upper panel). The middle panel shows the equivalent results for representations in the lower level, encoding the semantic *adjective* factor, which switches between green and red for the last three questions. The lower panel shows the band-pass filtered responses (between 4 and 32 Hz) to illustrate the nesting of simulated electrophysiological responses (solid lines: higher-level *scenic* responses. Broken lines: lower-level *semantic* responses). Two responses have been highlighted for illustration in red (high level) and cyan (lower level). The nesting of (simulated) neuronal fluctuations is evident at a number of levels. First, bursts of activity are organised around periods of belief updating, when sensory evidence becomes available. Periods of activity are evoked by auditory outcomes (words) at the lower level and—at the higher level—evidence that speaks to the posterior expectations or representations. Second, it demonstrates transients at the onset of each word, which recur at a theta frequency. Each transient carries fast (e.g., gamma) frequency components. This means there is a theta-gamma coupling in the sense that the amplitude of gamma responses fluctuates at a theta frequency. Finally, note that the transients at the lower level (cyan line) are ‘sharper’ than the transients at the higher level (red line).

The nesting of electrophysiological responses is illustrated in Fig. 9, which shows the simulated neuronal firing and associated local field potentials for neuronal populations at the higher and lower levels. Fig. 9A shows simulated unit responses at the upper level, Fig. 9B shows the same at the lower (semiotic) level, and Fig. 9C overlays simulated local field potentials at the upper and lower levels. The key thing to observe here is that lower level transients (cyan lines) are faster than the accompanying higher-level transients (red lines). This means that fluctuations in the amplitude of frequency-specific responses to each word or phrase will necessarily produce phase-amplitude coupling. Phenomenologically, this means that one would not be surprised to see bursts of beta activity at the higher level coincide with bursts of gamma activity in the lower-level. See (Arnal and Giraud, 2012; Giraud and Poeppel, 2012) for a discussion of related phenomena.

Crucially, from this perspective, amplitude-to-amplitude and phase-amplitude coupling are simply two ways of quantifying non-linear coupling that inherit from the nesting of transients under a hierarchical generative model. This nesting of transients would be interpreted as some form of nonlinear or phase-amplitude coupling, if subject to standard empirical analysis procedures such as bi-coherence analysis or phase-synchronisation measures: for example, see (Giraud and Poeppel, 2012; Lizarazu et al., 2019; Pefkou et al., 2017). In interpreting these effects, it is interesting to note a subtle distinction between phase-amplitude and amplitude-to-amplitude coupling in an idealised setting: the phase-amplitude coupling is a necessary result of the hierarchical modelling, because gamma-frequency updates are scheduled at the slower theta frequency. However, amplitude-to-amplitude coupling between theta and gamma results from departures from the perfect theta rate of gamma transients, because the amplitude of theta activity must vary to produce amplitude-to-amplitude coupling. As is evident in Figs. 8 and 9, both of these data features would have arisen under conventional analyses of the electrophysiological responses simulated here. Practically speaking, however, it is difficult to distinguish different types of cross frequency coupling in real data due to Heisenberg's uncertainty principle (e.g., see (Aru et al., 2015; Munia and Aviyente, 2019; Nakhnikian et al., 2016)). In other words, the apparent amplitude-to-amplitude coupling reflects the way that people quantify this type of coupling. For this reason, the phase-amplitude coupling is a more interesting feature of the hierarchical generative model we describe. In general, this nonlinear coupling is consistent with “evidence that the temporal modulation transfer function (TMTF) of human auditory perception is not simply low-pass in nature” (from (Edwards and Chang, 2013) p113).

### 4.2 Deep violation responses

Thus far, we have focused on the sort of message passing—and its neurophysiological correlates—that would be measured using time frequency analyses of induced responses. Here, we consider how the same computational architecture generates predictions for evoked responses. In particular, we show that stimuli that violate expectations generate differential (mismatch) waveforms, which have been the focus of many empirical studies; e.g., (Coulson et al., 1998; Friederici, 2011; Pulvermuller et al., 1995; Van Petten and Luka, 2012; Ylinen et al., 2016).

**Fig. 10:**
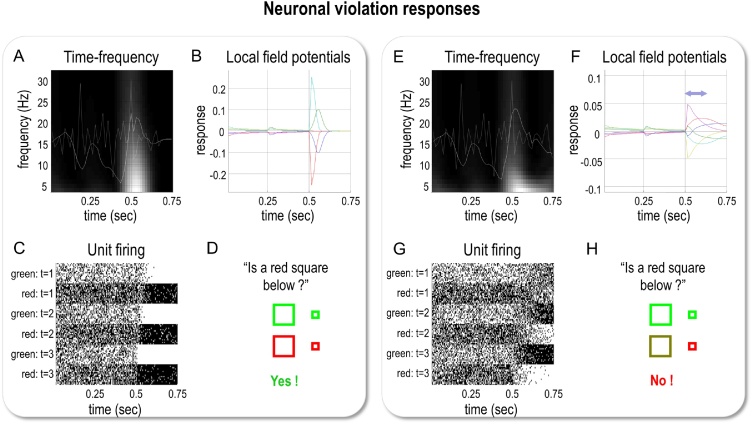
**Violation responses**: This figure illustrates the neurophysiological simulation of a violation response, of the sort seen in response to a semantic violation or unexpected sentence closure. We reproduced this paradigm by rerunning the fifth narrative but supplying the wrong answer at the end (see panel H). The left box (A–D) shows the standard responses when the correct answer is supplied (see panel D) using a similar format to Fig. 7. Here, the simulated unit firing of neurons that respond to the *colour* of the lower object (i.e., the *scenic* representation at the higher level) are shown in raster format (panel C). The population average or expected firing rate is used to simulate unit activity by sampling from a binomial distribution at each 16 ms time window. The average response magnitude and time frequency response are shown in panel A for the three epochs (*prompt*, *question*, *answer*) of the fifth exchange. The simulated event-related potentials (i.e., expectations about the colour of the lower object—red or green—at the three epochs, band pass filtered at 4–32 Hz) are shown in panel B. The right box (E–H) reproduces the same results after supplying the wrong answer (i.e., “No” versus “Yes”), which induces protracted belief updating over a longer latency, as indicated by the blue arrow.

Fig. 10 illustrates the neurophysiological simulation of a violation response; for example, P300 or N400 responses to a semantic violation or unexpected sentence closure. Here, we reproduce a violation paradigm by rerunning the fifth exchange from the previous simulations with the wrong answer at the end. Recall that at the beginning of fifth exchange, the agent is confident that the colour of the lower shape is red: it obtained this information from the answer to question 2. The left panels show the standard responses, using a similar format to Fig. 7. Simulated event related potentials (i.e., band pass filtered expectations of the lower colour at the three epochs) are shown on the upper right. The underlying unit activities producing these fluctuations are shown in terms of a simulated raster of unit firing—for the six units in question—on the lower left. Of note, simulated event related potentials in the right panel (i.e., when the wrong answer is provided at 0.5 s) have longer latencies (as illustrated by the blue arrow). These long latency responses have a remarkably similar morphology to P300 (Donchin and Coles, 1988; Van Petten and Luka, 2012) and N400 (Kutas and Hillyard, 1984; Van Petten et al., 1999; Van Petten and Kutas, 1990) waveform components in empirical violation paradigms. Here, they simply reflect the fact that the artificial agent has to change its mind and undo the conviction that the lower square was red, given the evidence was in favour of green; in other words, update its beliefs. As can be seen from the lower right panel, the agent becomes less certain about the colour, and leans slightly towards the belief that the colour of the lower object is green. This posterior belief is entirely congruent with hearing a negative answer to the question “is there a red square below?”.

## 5 Synthetic communication

In the simulations above, external states of affairs were used to supply veridical answers to the first four questions by sampling from the generative process. In other words, the external states were standing in for the beliefs of someone answering or asking questions. In what follows, we make an important move and replace external states with another synthetic subject. This has the interesting consequence of taking external states off the table: outcomes are generated or sampled from the (posterior) predictions of one or other subject, so at no point do we need to refer to (external) states of the world (see Fig. 1). This is a straightforward consequence of allowing agents to generate outcomes that are shared between them. Heuristically, the imperative to resolve uncertainty (i.e., minimise expected free energy) is now reflected in a synchronisation of belief states; namely, a ‘meeting of minds’ and mutual understanding.

### 5.1 Questions and answers

**Fig. 11:**
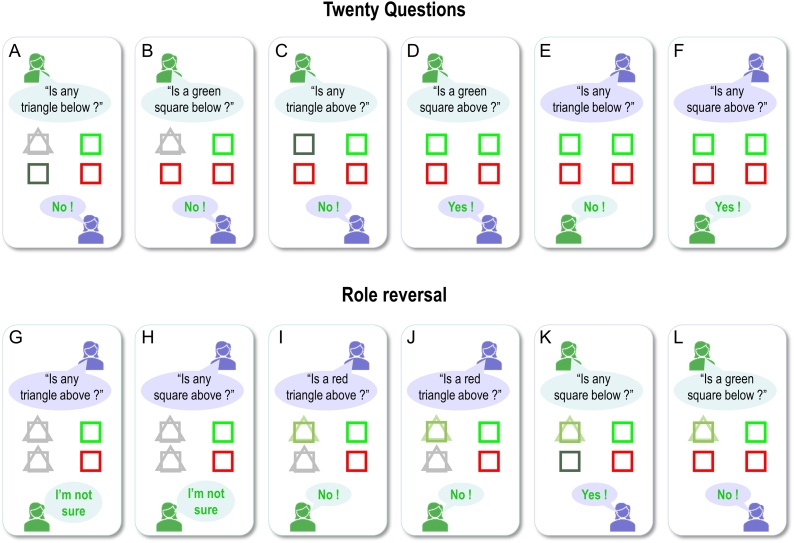
**Playing ‘Twenty Questions’ with a partner**: These simulations use a similar format to Fig. 6; however, here there are two synthetic subjects. Their beliefs are displayed in separate columns within each panel, and the text is placed next to the agent who spoke the phrase. The second subject (purple icon, right column) has precise (i.e., confident) beliefs about the scene at hand: it believes there is a green square above a red square). In contrast, the first agent (green icon, left column) begins with imprecise beliefs and effectively inherits the beliefs of the confident subject, by listening to the answers to the questions it asks. It is then able to answer the two questions asked by the other agent in the fifth and sixth narratives. The lower panels replicate the simulation but here the less confident agent answers questions.

The simulations reported in Fig. 11 use a similar format to Fig. 6; however, here there are two synthetic subjects. The second subject has precise (i.e., confident) beliefs about the scene at hand (namely, a green square above and a red square below). In contrast, the first subject is less confident before the exchange begins and effectively inherits the belief of the confident subject by listening to the answers to the questions it asks. Analogous to Fig. 6, after the fourth answer, the first subject has a precise understanding of what the confident subject believes and is able to answer correctly the when quizzed with two final questions.

In this example, the first agent chose its questions carefully to resolve as much uncertainty as possible. In the lower panel, we reverse the roles so that the second (confident) agent asks questions and the less confident agent provides answers. The results of the simulation are shown in the lower panel (entitled role reversal). In comparison to the upper panel, the first agent accumulates evidence for the beliefs of the second agent much more slowly, and the two agents do not share the same beliefs after the fourth answer. The questions asked by the second agent are insensitive to the particular uncertainty that confounds the first, and so all the first agent can do is say that it is “not sure” in response to the first two questions, when its beliefs are uniform across the *red* and *green* states. For the third question, it responds “no”. Unlike the first two questions, the third question asks about the combination of colour and shape attributes in the upper location, which has four possible options, and so the balance of probabilities means that the most likely answer is “no”. After hearing its own negative answer, the subject then convinces itself that the upper shape cannot be a red triangle and is more likely to be a green square, which is further endorsed by its subsequent response to the (same) fourth question. Only when observing definitive and veridical answers can it then start to accumulate proper beliefs about what the other subject believes.

### 5.2 Storytelling

**Fig. 12:**
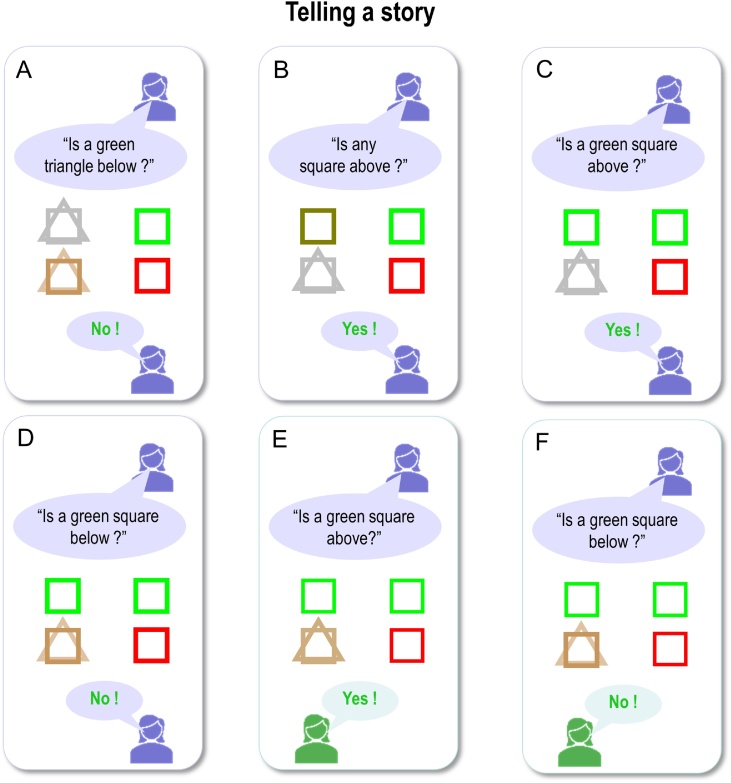
**Storytelling**: The result of an exchange between two synthetic agents, when the second agent (purple icon, right panel) answered its own questions for the first four exchanges (panels A–D). For the fourth and fifth exchanges (panels E–F), the second agent asked the questions and the first agent (green icon, left column) answered. Here, the first agent had to rely upon the question selected by the second agent to update its beliefs about the scene. This resulted in some residual ambiguity about the lower object (i.e., it is most likely to be a red triangle, it could be a red square, but it is probably not a green square). Nevertheless, the first subject was still able to answer the questions correctly.

We can use exactly the same scheme above to simulate instruction or storytelling: the same underlying joint belief updating characterises all forms of exchange in this active inference formulation. We reran the simulations from the previous section, but this time the second agent answered its own questions (Fig. 12), while the first simply listened for the first four exchanges and supplied answers for the last two exchanges. As above, the first agent inherits scenic beliefs from the second agent, but here this is simply by listening to the second agent’s soliloquy. After the four questions and answers, the first agent is sufficiently confident about the scene to answer correctly; even though it is unsure whether the lower object is a red square or a red triangle. This ambiguity reflects the fact that the preceding questions and answers were not selected to reduce *the first agent’s* uncertainty—they were selected by the second subject, who had very precise beliefs.

### 5.3 Making your mind up

**Fig. 13:**
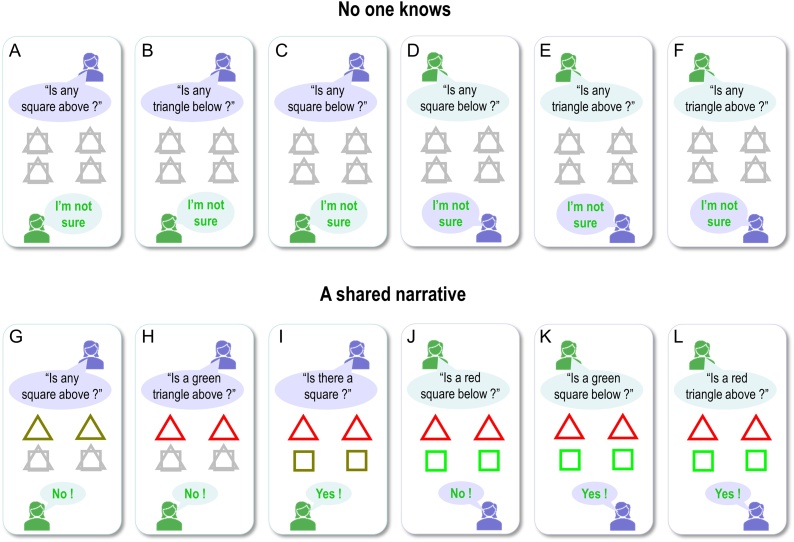
***Folie à deux***: The result of an exchange between two interlocutors (green and purple), who are both unsure about the scene they are discussing. The format of this figure follows that of previous figures. The upper panels (A–F) show the questions and answers that confess a lack of knowledge or certainty. Each agent’s posterior expectations about the scene are indicated by the coloured shapes. In this simulation, neither agent informs the other agent about the objects present in the scene, and so they both remain in a state of mutually consistent ignorance. The lower panels (G–L) show the same simulation when the likelihood of an “I’m not sure” response was set to zero. This produces a *folie à deux* described in the main text. In brief, the ensuing belief updating starts from an unstable fixed point of uncertainty that converges onto a shared fantasy about what both agents (are confident they) believe.

Things get interesting if we reduce the precision of prior beliefs, so that both subjects are uncertain about the scene. Recall that the synthetic agents are given three possible responses: “Yes!”, “No!”, and “I’m not sure”. When allowed to question each other in this setting, they simply respond truthfully that they are unsure about the answer (see the upper panel of Fig. 13). However, when we reduce the prior probability of the ‘not sure’ response, both subjects effectively tell each other about what they believe, until they come to hold the same beliefs (see the lower panel of Fig. 13). At this point, uncertainty is precluded because each can predict the other and their shared understanding. This is an example of neural hermeneutics (Frith and Wentzer, 2013) in the absence of ‘truth pointing’. As noted above, this is a form of generalised synchronisation (Friston and Frith, 2015a), where the orbits of belief states that underlie linguistic exchange become mutually predictable as (expected) free energy is minimised. Anthropomorphically speaking, the two synthetic subjects have simply reached a consensus about how to describe some shared construct. Crucially, the construct (i.e., scene) does not exist and, from our perspective, therefore, could be described as a *Folie à deux* (Arnone et al., 2006). On a more positive note, it could also be construed as a joint exercise in creative thinking. Although not pursued here, one can think about extensions of this sort of simulation that could be framed in terms of artistic communication and creativity, bringing us back to the resolution of uncertainty through epistemic foraging, novelty and fun (Schmidhuber, 2006).

Returning to the upper panel of Fig. 13, this example illustrates the joint maintenance of uncertain beliefs. This is interesting because these rudimentary agents have no formal metacognitive capacity (see discussion). In other words, their uncertainty is implicit in neuronal states encoding uncertain belief distributions, rather than possessing neuronal states that encode posterior beliefs about the precision of their beliefs. Having beliefs about the precision of beliefs may sound rather complicated; however, statistical models with a deep structure very often encode uncertainty explicitly. For example, when we report the degrees of freedom of a statistical test, we are effectively reporting the confidence in our estimate of uncertainty; e.g., the standard error on some parameter estimates (Friston et al., 2007). In the current simulations, there is no such metacognitive inference—and yet the two agents continue to answer that they are uncertain about the hidden states they are being questioned on, as is Bayes optimal.

The mechanism that underwrites this apparent confirmation of ‘known unknowns’ is straightforward. It rests upon a nontrivial likelihood of saying “I'm not sure”, irrespective of one's beliefs. Consider the following: I am thinking about numbers between one and one hundred and I can either report a number or select a “not sure” option. If the likelihood of reporting a number is 90 % and I am sure about the number, then I am nine times more likely to report the (exact) number I have in mind than to say “not sure”. Conversely, if I have no idea about the number, then the likelihood of reporting any number is equal to the probability of selecting any other number; the probability of reporting any individual number therefore falls to less than 1 %, because the probabilities are dispersed or *diluted* over 100 number options. In this case, I am therefore more than 10 times more likely to report “not sure” than any individual number. In Bayesian model selection, this phenomena is known as *evidence dilution* (Hoeting et al., 1999). The example in the upper panel of Fig. 13 highlights this emergent but simple consequence of entertaining declarations of uncertainty. Note that this kind of uncertainty rests upon a shared generative model, in which uninformative responses can be selected, even in the absence of uncertainty. When we remove the opportunity to generate such agnostic responses, a different pattern of mutual understanding emerges (see the lower panel of Fig. 13).

## 6 Discussion

In summary, we have illustrated a number of plausible correlates of communication that emerge from active inference under a particular sort of generative model. This generative model was motivated by the role of language in communicating a narrative. The key attributes of this model speak to the notion of a shared narrative that reduces uncertainty. In Section 4 (“Twenty Questions” simulations), we simulated an agent that was speaking to itself (i.e., in ‘conversation’ with a generative process), and in Section 5 (Synthetic communication) we used exactly the same generative model to simulate two subjects who were asking and answering questions. In each of these cases, the inference and sensory evidence were identical: the only difference was agency (i.e., who was talking). Generally speaking, these simulations demonstrate that the beliefs of two synthetic agents converge, even when they initially had different prior beliefs. This simply reflects the fact that an agent updates its beliefs based on answers (i.e., observations) from the other agent—and would therefore generalise to other situations where the prior beliefs of two synthetic agents differ.

In our simulations, hierarchical inference led to belief updating that resembled theta-gamma phase-amplitude coupling (Fig. 8), which has often been observed empirically in studies of speech perception; for example, see (Giraud and Poeppel, 2012; Lizarazu et al., 2019; Pefkou et al., 2017). Of relevance, our simulated neuronal responses reflect belief updating under model inversion—corresponding to speech perception rather than production. Under this framework, phase-amplitude coupling between theta and gamma frequencies arises because hearing each word induces a succession of transients (i.e., belief updates) containing high-frequency (gamma) components, and these transients recur at the frequency of word presentation, which is in the theta range. This produces simulated responses that would be interpreted as phase-amplitude coupling. A multi-timescale nesting process has been proposed by others as a plausible explanation (Arnal and Giraud, 2012; Giraud and Poeppel, 2012), as it has been noted that the timing of these rhythms corresponds to important timescales in language. Previous approaches to modelling this phenomenon (Hovsepyan et al., 2018; Hyafil and Cernak, 2015) have been data-driven—incorporating explicit theta and gamma ‘units’. Here, we took a theoretical approach and show that theta-gamma coupling can arise from belief updating, given an agent’s goal to understand the contents of a scene from a dialogue.

Our simulations also predict electrophysiological violation responses, of the sorts observed in P300 and N400 studies. The P300 has been observed in oddball paradigms, in which repeating stimuli are interspersed with unexpected deviants. In this setting, the P300 has been interpreted as reflecting violations of high-level context (Donchin and Coles, 1988). The N400 is commonly observed in studies of language. It has been elicited when participants hear words that have low frequency (Kutas and Hillyard, 1984; Van Petten et al., 1999; Van Petten and Kutas, 1990), or words that are semantically related to words that have high probability (Kutas and Hillyard, 1984). These types of mismatch waveforms have been demonstrated in a previous application of active inference to speech perception (Friston et al., 2020). Here, we demonstrate that a hierarchical model capable of generating these types of mismatch responses is also capable of simulating theta-gamma coupling. In previous work, we showed a distinction in the ERPs generated at different levels of a hierarchical generative model in a local-global paradigm (Friston et al., 2017d). In future work, it would be interesting to use the current generative model to simulate violations at different levels of the hierarchy in a similar way, and compare these to empirical data showing that different types of violations generate distinct ERPs (for examples, see Connolly and Phillips, 1994; Osterhout et al., 1996).

The generative model we have introduced represents a different way to think about semantic or contextual aspects of language, in relation to previous accounts. Surveying the empirical and theoretical antecedents of the current formulation of language—and understanding—would be an enormous undertaking, given the vast amount of psychological, philosophical and computational literature in this area. In this context, three observations are relevant. First, in the current framework, belief updating is hierarchical: beliefs about the content of a scene are maintained at the higher level. Second, an agent’s *uncertainty* in their beliefs about the current state of the world affect the magnitude of belief updating. Finally, here, we cast language understanding as an active processes—allowing an agent to ask questions that maximally resolve their uncertainty about states of affairs. Although the finer details of the states in the model are somewhat simplified, our aim was to provide a general computational architecture that can be used to simulate basic linguistic communication.

Before commenting upon some salient points of contact with related work, we will qualify this discussion with the following observations: if one commits to active inference (and implicitly, the free energy principle), there is little latitude for hypothesising about the nature and form of linguistic processing. This is because everything of interest is defined operationally by the generative model and the generative model is, in turn, defined by what we want to explain; namely linguistic communication. In other words, simply defining the inference problem dictates the form of the requisite generative model, in terms of what how outcomes are caused by states of the world (or others). Furthermore, once the generative model has been specified, the belief updating is prescribed by standard belief updating schemes; here, variational or marginal message passing (Dauwels, 2007; Parr et al., 2019b; Winn and Bishop, 2005).

This means that there is no latitude to accommodate alternative hypotheses or constructs, if they are not consistent with the sort of formulation above—or the basic architecture of belief updating. In short, in active inference, the only questions are: what kind of generative model could explain these responses? Strictly speaking, this precludes questions about the implementation and the neurophysiological correlates of language processing (Braiman et al., 2018; Dowty et al., 1985; Lizarazu et al., 2019; Pefkou et al., 2017; Wilson et al., 2017; Ylinen et al., 2016). While many of these may be especially useful within their own remit, unless neurophysiological correlates can be linked to belief updating (i.e., understanding through communication), they cannot be used to simulate—and therefore understand—communication. In a similar vein, any exciting advances in computational neurolinguistics (Barlow, 1974; Lightfoot, 1979; MacKay and Peto, 1995; Norris et al., 2016; Rosenfeld, 2000) that do not deal explicitly with belief states updating cannot be used to create artefacts that communicate. For example, the use of deep learning in speech recognition may provide compelling insights into the computational architecture of linguistic processing at an auditory level; however, speech recognition does not constitute understanding. In other words, simply mapping from auditory input to a list of words does not constitute the inversion of a generative model. Some research, within machine learning, has looked at schemes similar to active inference, within partial observability frameworks. For example, the Bayesian Action Decoder (Foerster et al., 2018) uses approximate Bayesian update to obtain a ‘public’ belief that is conditioned on the actions of all agents in the environment, leading to efficient communication when playing multi-agent games.

In this paper, we specified outcomes as words rather than an acoustic timeseries, because the mapping from words to acoustics has already been considered from the perspective of active listening (Friston et al., 2020). This allowed us to focus on aspects of the generative model that are specific to language and communication. Combining the current model with active listening (Friston et al., 2020)—which maps between words and the acoustic timeseries—would allow future work to systematically investigate other factors influencing spoken communication, such as the influence of noise. Although we have framed the current work in terms of speaking and listening, we note that—in its current form—it also applies to written communication, such as the exchange of text messages.

There are important developments in computational linguistics that could inform active inference schemes in a useful way. For example, the use of hierarchical Dirichlet processes to solve the structure learning problem in generative models of language (MacKay and Peto, 1995; Salakhutdinov et al., 2013) could be the right approach to grow generative models—and subsequently prune them with Bayesian model reduction (Friston and Penny, 2011)—in the context of language acquisition. We have not touched upon this issue in the current paper; however, having established the basic form of a generative model for language and understanding, the next challenge would be to study learning through optimisation of the model parameters; e.g., the likelihood mapping is entailed by the **A** matrices between hierarchical levels. After this (learning) has been addressed, the next level of optimisation concerns the form and structure of the model itself. For example, how many hidden factors should be included—and how many levels or mutually exclusive states occupy each factor? This is the problem of structure learning (Catal et al., 2019; Gershman, 2017; Tenenbaum et al., 2011; Tervo et al., 2016) that is elegantly addressed using nonparametric Bayesian methods (Collins and Frank, 2013; Goldwater, 2006; Teh et al., 2006), such as those found in computational linguistics (please see below). Importantly, the hidden factors within the generative model are factorised and, therefore, the belief updating in the current paper should be preserved if additional factors were wadded. Adding additional factors only becomes interesting if they interact with other states to affect outcomes—in which case, the current framework would allow the behavioural and neurophysiological consequences of these interactions to be estimated. Similarly, simply adding additional mutually exclusive states within a factor would not affect inference unless they engender high probability policies within Occam’s window—in which case, belief updating may be slower. Questions about the structure of the generative model would be interesting topics for future work.

At the lower level, we factorised syntax and semantics into separate factors. This was intuitive for the current application, in which different syntax could be used to ask questions about the same features of the scene (i.e., shape, colour, and position). We acknowledge there is a long-standing debate as to whether syntax and semantics are independent (e.g. Dick et al., 2001; Kuperberg et al., 2003; Siegelman et al., 2019), and extensions of this model may wish to consider this aspect more carefully. One advantage of this framework is that competing hypotheses about the structure of the model can be compared using Bayesian model selection (Stephan et al., 2009). In other words, this would allow researchers to test whether the best explanation for their data is a factorisation of syntax and semantics or some alternative with a more nuanced dependency structure.

In this paper, we ignored the attribution of (i.e., inference about) agency; namely, metacognitive capability (Fleming et al., 2012; Shea et al., 2014). This means that each synthetic subject had no notion of who was talking, and the ‘turn taking’ in our simulations needed to be handcrafted. Nevertheless, our synthetic subject could still use the information provided to resolve uncertainty about states the world (e.g., the configuration of objects in a scene). More sophisticated generative models would include hidden factors that include agency *per se*. This was not necessary for the current examples, but would be necessary for simulating turn taking in linguistic exchange (Garrod and Pickering, 2009; Ghazanfar and Takahashi, 2014; Wilson and Wilson, 2005). This was a focus of our earlier work using simulated songbirds (Friston and Frith, 2015a). In the current work, we simply replaced internally generated speech with the external speech of a conversant to simulate asking questions and answering, respectively. However, the agents were not aware of this.

An important aspect of metacognition is knowing when one is uncertain. In the simulations above, agents were able to maintain their uncertainty by providing each other with uninformative (“not sure”) answers. However, they were not aware of being uncertain (i.e., their generative models did not have a hidden ‘state of uncertainty’). A more sophisticated generative model would realise that something was not known with confidence and respond with "I really don't know". This apparently simple capacity rests upon a generative model of confidence that is quintessentially metacognitive; in the sense that inverting this kind of deep generative model produces (posterior) beliefs about beliefs.

It is an interesting challenge to formulate metacognitive depth using discrete state space models (i.e., hidden Markov models or Markov decision processes). In one sense, the encoding of precision or confidence in beliefs about policies is a metacognitive representation (see the simulated dopamine responses in Fig. 7); however, it is quite elemental. Furthermore, this sort of representation is a continuous (real valued) variable, of the sort that has been used to explain dopaminergic fluctuations in reinforcement learning paradigms (Schwartenbeck et al., 2015). It would be nice to have the categorical step state of “I am uncertain” or “I am very confused”. This speaks to the use of higher hierarchical levels that prescribe uniform (empirical) priors over the initial states of a level below. In other words, one can generate belief distributions about the context of a lower level, based upon a discretisation into confident beliefs about particular states of affairs and complete uncertainty (with uniform priors). In principle, this should equip agents with a metacognitive sense of their beliefs—and a way of communicating these beliefs via language.

An important aspect of language that we ignored is its computational richness (e.g., discrete infinity) afforded by the combinatorics of narratives and sentences (Chomsky, 2017). In addition, we have ignored the parsing and transpositions that characterise real language processing—that themselves have a deep hierarchical form. This issue presents some interesting challenges, in terms of articulating the structure of the generative model, which may involve separately generating the ordinal aspects of spoken language from its content. Technically, this would involve an interaction between—or coupling of—separate ordinal and content factors (Dehaene et al., 2015; Friston and Buzsaki, 2016). In other words, we would have to replace the probability transition matrices (**B**) above with high dimensional arrays, so that the probability transitions among the levels of one factor depend upon the level of another. Note that learning the factorial structure of natural language is the focus of much work: e.g., neural language modelling using recurrent neural networks (Bengio et al., 2003; Mikolov, 2010; Mikolov et al., 2013; Shang et al., 2015), or sequence-to-sequence modelling (Bahdanau et al., 2014; Ghazvininejad et al., 2018; Sutskever et al., 2014; Vinyals and Le, 2015).

We have not considered language acquisition; e.g. via the learning of the **A**, **B** and **D** parameters above (Al-Muhaideb and Menai, 2011; Bengio et al., 2009; Friston et al., 2016). In principle, by listening to an authoritative sequence of questions and answers, it should be possible to simulate language acquisition at various levels, via structure learning and Bayesian model reduction (Tervo et al., 2016). This has been pursued in the context of abstract rule learning (Friston et al., 2017b), but has not been applied in the present context. At this point, we get close to the problems addressed in computational linguistics, via the use of hierarchal Dirichlet processes (MacKay and Peto, 1995; Salakhutdinov et al., 2013; Teh et al., 2006). In this setting, the key problem is to optimise the structure and hierarchal form of the model—and to know when to add an extra factor or level. It is possible that this structure learning problem may be usefully addressed with existing work on hierarchal Dirichlet process models and nonparametric Bayes (Goldwater, 2006); combined with the more top-down approach promoted in this work.

Finally, our syntax factor is over-simplistic, encompassing only a handful of possibilities. This was sufficient for the simulations we presented, but will become important in applications of this kind of generative model. There is a substantial literature on cognitive models of syntax processing (for a recent review, see Demberg and Keller, 2019) and how listeners deal with semantic ambiguity (Altmann and Steedman, 1988; Bever, 1970; Gibson, 1998, 2000). Generally speaking, evidence from visual paradigms (Kamide et al., 2003) points to a predictive process, which is broadly consistent with active inference. It has also been proposed that syntax may itself be hierarchical (Van Schijndel et al., 2013).

In summary, we have presented a generative model and inference scheme that is capable of simulating exchanges between synthetic subjects. This generative model is deep and hierarchical: inferences at the higher level inform words that are selected at the lower level—and these levels are nested, such that phrase-level inferences generate the words contained within the phrase, and then the lower level ‘resets’ for the next phrase. Our simulations of the “Twenty Questions” game show that agents can select the best questions—to ask of another—to reduce their uncertainty (in a Bayes optimal fashion) about the subject of conversation. We have also shown that, if the agent has precise beliefs about the nature of the scene, it can correctly answer another agent’s questions. These types of exchanges demonstrate a convergence of beliefs, reflecting a successful linguistic exchange. We have also simulated situations where, if the agent has very imprecise beliefs, it will acknowledge its own uncertainty. If two agents both start with imprecise beliefs, then their generative models will converge, even though neither agent knows the veridical state of the scene. This type of setting could be considered as a *folie à deux* or an example of joint creative thinking. Finally, this formulation of communication makes predictions for neurophysiological responses, based on belief updating. It predicts violation responses, like P300 and N400 responses, when an answer is inconsistent with the agent’s beliefs, and shows theta-gamma coupling as an emergent property of belief updating. Overall, we envisage that this model will be a useful starting point for simulating more complex linguistic exchanges—that include metacognition, or which simulate language acquisition.

## 7 Software note

Although the generative model changes from application to application, the belief updates—and simulated neuronal responses—described in this paper are generic and can be implemented using standard routines (here **spm_MDP_VB_X.m**). These routines are available as Matlab code in the SPM academic software: http://www.fil.ion.ucl.ac.uk/spm/. The simulations in this paper can be reproduced (and customised) via a graphical user interface by typing **DEM** and selecting the **20 questions** demo.

## Declaration of Competing Interest

None.

## Appendix A – *Expected free energy*: the variational free energy of a policy is a functional of an approximate posterior distribution over states Q(s|π) given observed outcomes *o*, under a probabilistic generative model P(o,s|π), given a policy π

(A.1) $$\begin{array}{c} F=E_{Q(s|\pi )}[\ln Q(s|\pi )-\ln P(o,s|\pi )]\\ =\underset{\text{complexity}}{\underset{︸}{D_{KL}[Q(s|\pi )||P(s|\pi )]}}-\underset{\text{accuracy}}{\underset{︸}{E_{Q(s|\pi )}[\ln P(o|s)]}} \end{array}$$

The second equality expresses free energy as the difference between a Kullback-Leibler divergence (i.e., *complexity*) and the expected log likelihood (i.e., *accuracy*), given (observed) outcomes.

In contrast, the expected free energy of a policy is an average over (unobserved) outcomes that determines the distribution over (future) states:

(A.2) $$\begin{array}{c} G=E_{Q(o,s|\pi )}[\ln Q(s|\pi )-\ln P(o,s|\pi )]\\ =E_{Q(o,s|\pi )}[\ln Q(s|\pi )-\ln P(s|o,\pi )-\ln P(o|\pi )] \end{array}$$

Replacing the true predictive posterior with the approximate predictive posterior gives:

(A.3) $$\begin{array}{c} G=E_{Q(o,s|\pi )}[\underset{\text{intrinsic value}}{\underset{︸}{\ln Q(s|\pi )-\ln Q(s|o,\pi )}}-\underset{\text{extrinsic value}}{\underset{︸}{\ln P(o|\pi )}}]\\ =\underset{\text{risk}}{\underset{︸}{D_{KL}[Q(o|\pi )||P(o|\pi )]}}+\underset{\text{ambiguity}}{\underset{︸}{H[Q(o|s)]}} \end{array}$$

The first equality expresses expected free energy in terms of *intrinsic* and *extrinsic value*, while the second expression is an equivalent formulation in terms of the divergence between predicted and prior preferences over outcomes (i.e., *risk*) and expected uncertainty about outcomes, given their causes (i.e., *ambiguity*). By comparing Eq. A.3 with A.1, it can be seen that *risk* is expected *complexity* and *ambiguity* is expected *inaccuracy*, under a particular policy.

Note that, for completeness and clarity, in the above equations (and the expressions in Table 1) we have conditioned prior preferences on policies. In practice, however, we assume that prior preferences do not depend upon policies.

## Appendix B – *Belief updating*: approximate Bayesian inference corresponds to minimising variational free energy, with respect to the sufficient statistics that constitute posterior beliefs. For generative models of discrete states, the free energy of hidden states and policies can be expressed as the (time-dependent) free energy under each policy plus the complexity incurred by posterior beliefs about (time-invariant) policies, where (with some simplifications)

$$\begin{array}{c} F[Q]=D_{KL}[Q(s,\pi )||P(s,\pi )]-{\text{E}}_{Q}[\ln P(o|x)]\\ =\sum_{\tau }{\text{E}}_{Q}[F(\pi ,\tau )]+D_{KL}[Q(\pi )||P(\pi )]\\ =\pi \cdot (\ln\pi +F+G) \end{array}$$

The free energy of hidden states under each policy is then given by:

(B.4) $$\begin{array}{c} F_{\pi }=\sum_{\tau }F(\pi ,\tau )\\ F(\pi ,\tau )=\underset{\text{complexity}}{\underset{︸}{E_{Q(s_{\tau -1}|\pi )}[D[Q(s_{\tau }|\pi )||P(s_{\tau }|s_{\tau -1},\pi )]]}}-\underset{\text{accuracy}}{\underset{︸}{E[\ln P(o_{\tau }|s_{\tau })]}}\\ =s_{\pi ,\tau }\cdot (\ln s_{\pi ,\tau }-\ln B_{\pi ,\tau -1}s_{\pi ,\tau -1}-\ln A\cdot o_{\tau }) \end{array}$$

The expected free energy of a policy has a similar form, but the expectation is over hidden states and outcomes that have yet to be observed; namely, $Q(o_{\tau },s_{\tau })=P(o_{\tau }|s_{\tau })Q(s_{\tau }|\pi )$.

(B.5) $$\begin{array}{c} G_{\pi }=\sum_{\tau }G(\pi ,\tau )\\ G(\pi ,\tau )=-\underset{\text{intrinsic value}}{\underset{︸}{E_{Q}[\ln Q(s_{\tau }|o_{\tau },\pi )-\ln Q(s_{\tau }|\pi )]}}-\underset{\text{extrinsic value}}{\underset{︸}{E_{Q}[\ln P(o_{\tau }|\pi )]}}\\ =\underset{\text{risk}}{\underset{︸}{D_{KL}[Q(o_{\tau }|\pi )||P(o_{\tau }|\pi )]}}+\underset{\text{ambiguity}}{\underset{︸}{H[Q(o_{\tau }|s_{\tau })]}}\\ =o_{\pi ,\tau }\cdot (\ln o_{\pi ,\tau }+C_{\tau })+H\cdot s_{\pi ,\tau }\\ H=-diag(A\cdot \ln A)\\ C_{\tau }=-\ln P(o_{\tau }|\pi ) \end{array}$$

Please see Table 1 for a definition of the variables in these equations.
